# A population-based study of obstructive sleep apnea: using probabilistic case ascertainment through health administrative databases

**DOI:** 10.1093/ije/dyag176

**Published:** 2026-08-20

**Authors:** Robert Talarico, Marcus Povitz, Sachin R Pendharkar, William Reisman, Daniel I McIsaac, Tetyana Kendzerska

**Affiliations:** Ottawa Hospital Research Institute Clinical Epidemiology Program, Ottawa, ON, Canada; Institute for Clinical Evaluative Sciences, ICES Ottawa, Ottawa, ON, Canada; Department of Medicine, University of Calgary Cumming School of Medicine, Calgary, Canada; Department of Medicine, University of Calgary Cumming School of Medicine, Calgary, Canada; Department of Community Health Sciences, University of Calgary Cumming School of Medicine, Calgary, Canada; Department of Medicine, University of Western Ontario Schulich School of Medicine & Dentistry, London, Canada; Ottawa Hospital Research Institute Clinical Epidemiology Program, Ottawa, ON, Canada; Institute for Clinical Evaluative Sciences, ICES Ottawa, Ottawa, ON, Canada; Department of Anesthesiology & Pain Medicine, University of Ottawa Faculty of Medicine, Ottawa, Canada; Ottawa Hospital Research Institute Clinical Epidemiology Program, Ottawa, ON, Canada; Institute for Clinical Evaluative Sciences, ICES Ottawa, Ottawa, ON, Canada; Department of Medicine, University of Ottawa Faculty of Medicine, Ottawa, Canada

**Keywords:** obstructive sleep apnea, population-based, prevalence, bootstrap imputation approach

## Abstract

**Background:**

Although polysomnography (PSG) is the diagnostic gold standard, PSG data on obstructive sleep apnea (OSA) presence and severity are rarely available at the population level. Health administrative databases offer broad coverage for OSA case ascertainment but require methods that address inherent misclassification. We utilized a previously validated probabilistic case ascertainment model of OSA to estimate population-level prevalence and correlates of OSA.

**Methods:**

We conducted a population-based retrospective cohort study in Ontario, Canada. First, using a clinical cohort with PSG-confirmed OSA severity (*measurement cohort*; *n* = 18 581), we quantified misclassification in probabilistic OSA case definitions. We then applied the externally validated probability models to all adults 18 years and older in Ontario who underwent in-laboratory overnight PSG between 2010 and 2018 (*provincial PSG cohort*; *n* = 834 361). OSA prevalence and correlates were estimated using multiple probability thresholds and a threshold-free bootstrap imputation approach (BIA).

**Results:**

Compared to the PSG-based gold standard, the BIA surrogate definition had the lowest average relative bias in OSA prevalence estimation (BIA: 5% versus >38% probability threshold: 11.4%). Using BIA, we estimate a 44.3% prevalence of moderate-severe OSA in the provincial PSG cohort, which in scenario-based projections yields a 3.4%–17.1% general population prevalence dependent on an assumed OSA underdiagnosis rate. OSA prevalence was higher in men, older ages, and previous comorbidity and was stable annually.

**Conclusion:**

Probabilistic case ascertainment using health administrative data enables robust population-level estimation of OSA epidemiology despite the absence of clinical diagnostic data and provides a framework for studying OSA prevalence, correlates, and outcomes over time.

Key Messages

- Misclassification bias is pervasive in obstructive sleep apnea research using routinely collected health data, and innovative probabilistic case ascertainment methods have potential.
- This study exemplified that a threshold-free bootstrap imputation approach minimized bias relative to polysomnography and yielded an estimated 44% prevalence of moderate-severe OSA among those undergoing sleep studies, corresponding to a general population prevalence of 3.4%–17.1% depending on underdiagnosis rates.
- These findings demonstrate that probabilistic case ascertainment methods can reduce misclassification bias to enable robust population-level surveillance of OSA and provide a generalizable framework for studying disease burden, correlates, and outcomes using routinely collected health administrative data.

## Introduction

Obstructive sleep apnea (OSA) is the most prevalent sleep-disordered breathing condition, affecting up to one billion adults worldwide [1] and representing a growing population-level burden [2]. OSA is a modifiable risk factor for adverse health outcomes and increased healthcare utilization [3–5]. Although substantial evidence exists on the association between OSA and increased all-cause and cardiovascular mortality [5, 6], its impact on other health outcomes and population prevalence is inadequately characterized. Large population-based cohorts are necessary to better quantify the determinants and distribution of OSA and inform healthcare planning and system-level coordination of sleep care.

OSA presence and severity are poorly captured using population-based health administrative databases (HAD) [7]. Prior research demonstrates that HAD-based OSA definitions use diagnostic or procedure codes with low sensitivity (<20%) and high specificity (91%–98%) [8, 9]. We previously derived and validated probability models to estimate OSA severity using patient-level characteristics from routinely collected HAD [10]. Applying a default >50% probability threshold improved sensitivity to ∼60% but reduced specificity below 90% [10]. Despite these gains, OSA misclassification remains pervasive.

Probabilistic case ascertainment requires translating predicted probabilities into disease status using a threshold. However, guidance on selecting optimal probability thresholds for probabilistic case definitions remains limited. A default 50% cutoff is commonly applied [11] but lacks empirical justification for conditions with low or moderate prevalence (<50%), such as OSA, and may introduce additional misclassification. Accordingly, threshold-free approaches have been developed and applied in HAD [12–14]. This method uses bootstrap imputation of disease status based on a validated prediction model.

Thus, we aimed to evaluate how probabilistic case-ascertainment models should be operationalized in population-based research, using OSA as an exemplar. Specifically, we sought to (i) quantify misclassification across alternative probability-based definitions using polysomnography (PSG) as the reference standard and (ii) estimate population-level OSA prevalence and examine associated sociodemographic and clinical correlates.

## Materials and methods

### Study design

We conducted a population-based retrospective cohort study in Ontario, Canada. This study applied previously developed and validated probabilistic OSA case-ascertainment models [10] to evaluate alternative probability-based definitions and generate population-level estimates.

First, we used a clinical cohort with PSG-confirmed OSA severity (*measurement cohort*) to quantify misclassification from different probability-based OSA case definitions. We then applied the validated probability models to all adults aged ≥18 years in Ontario who underwent in-laboratory PSG between 2010 and 2018 (*provincial PSG cohort*), within a universal healthcare system, where PSG is captured in HAD. Finally, we used the alternative OSA definitions to generate population-level estimates of OSA prevalence and association with known correlates. Reporting followed the STROBE guidelines and the RECORD extension for studies using HAD (Supplementary Table S7).

### Data sources

#### Clinical data (prior model development cohorts)

Previously, we developed and externally validated probabilistic OSA case-ascertainment models using two academic sleep-center cohorts with PSG data linked to HAD [10]. Further details are provided in Supplementary Table S1. These cohorts are used in the present study as the measurement cohort to evaluate misclassification and compare alternative definitions.

#### Health administrative data

Ontario provides universal health coverage and maintains comprehensive, linkable HADs using unique encoded identifiers at ICES (formerly known as the Institute for Clinical and Evaluative Sciences), an independent research institute. Further details on the specific databases are provided in Supplementary Table S2.

### Study populations

#### Measurement cohort

We combined the two clinical cohorts into a single measurement cohort, in which the apnea–hypopnea index (AHI), the gold standard for OSA presence and severity, was available for all patients and defined moderate-severe (AHI ≥ 15) and severe OSA (AHI > 30). Using the previously validated models [10], we generated fixed predicted probabilities of moderate-severe and severe OSA for all individuals in this cohort from linked HAD.

#### Provincial PSG cohort

We created a population-based cohort including all adults (≥18) in Ontario who underwent diagnostic PSG between 2010 and 2018. We ended follow-up in 2018 to allow for a 1-year observation period before the COVID-19 pandemic, which altered sleep service utilization [15]. For individuals with multiple PSGs, we retained the first. For this cohort, PSG parameters (e.g. AHI) were unavailable; only the date and type of PSG.

### OSA case ascertainment

In our prior study, the probabilistic models demonstrated strong discrimination, with externally validated c-statistics of 0.81 for moderate-severe OSA and 0.80 for severe OSA [10]. External calibration was superior for moderate-severe compared with severe OSA (Integrated Calibration Index: 0.02 versus 0.11) [10]. Model covariates, derived from HAD, included age, sex, sleep health services within 1 year post-index PSG (repeat PSG, OSA diagnosis code billed by an ADP-registered physician and PAP claim) and comorbidities in the preceding 3 years (hypertension, diabetes, and cardiovascular disease).

In the present study, we generated fixed model-based predicted probabilities of moderate-severe and severe OSA, ranging from 0 to 1, for individuals in both the measurement and the provincial PSG cohorts. We transformed these probabilities into three alternative case definitions for each severity level, as described below (Supplementary Table S2).

### Covariates

We utilized previously published approaches to define covariates [10]. We captured sociodemographic variables (neighborhood income and rurality) and calendar year of the index PSG. We used validated algorithms to capture prevalent chronic conditions prior to the index PSG, including chronic obstructive pulmonary disease, diabetes, congestive heart failure, hypertension, arrhythmia, and stroke [16–20], and calculated the Charlson Comorbidity Index through hospital records 3 years pre-index [21].

### Statistical analysis

Descriptive statistics summarized the cohorts, with standardized differences used for comparison. With the measurement cohort, we estimated OSA prevalence and computed crude relative risks (RRs) to identify OSA correlates. These estimates were based on the PSG-defined gold standard and were compared with alternative OSA definitions derived from model-based probabilities. To evaluate performance, we quantified relative bias in overall prevalence, subgroup prevalence, and covariate RRs. To summarize across covariates, we computed arithmetic means of prevalence, RR, and relative bias. Relative bias was defined as the absolute difference between each surrogate definition and the gold standard, divided by the gold standard (expressed as a percentage).

We converted the predicted OSA probabilities into three surrogate OSA definitions. First, we applied the Youden Index [22], which maximizes the sum of sensitivity and specificity, to classify OSA status (Present: yes versus no) (Supplementary Tables S3 and S4). Second, recognizing that optimal thresholds may differ for absolute (prevalence) versus relative (RR) measures, we identified thresholds that minimized average relative bias in prevalence (“Optimal Prevalence Threshold”) and in RRs across covariates (“Optimal RR Threshold”), based on the multidimensional bias analysis approach [23]. Third, we used a validated bootstrap imputation approach (BIA) [12, 13]. Briefly, in keeping with previous research and managing computational resources, 1000 bootstrap samples of the measurement cohort were drawn, and OSA status was imputed by comparing each individual’s predicted probability with a random draw from a uniform (0,1) distribution (Supplementary Table S8) [12–14]. Prevalence and RRs were estimated across resamples, with the median reported as the point estimate and the 2.5th and 97.5th percentiles as 95% confidence intervals (CIs). Finally, for each threshold-based definition, we applied a simple quantitative bias analysis (QBA) correction [23] to the prevalence and RR estimates using overall and covariate-specific specificity and sensitivity (Supplementary Tables S5 and S6). Unlike BIA, the QBA method does not use individual-level data; instead, it adjusts summary-level estimates based on external misclassification parameters.

We applied these methods to the provincial PSG cohort to estimate the prevalence of moderate-severe and severe OSA overall, by age, sex, and time. We estimated crude RRs and 95% CI of the selected covariates using a quasi-Poisson regression model. Given the high prevalence of undiagnosed OSA, we used the provincial PSG cohort to project prevalence in the entire adult Ontario general population (regardless of PSG status). We obtained a scenario-based lower bound (projected minimum) by multiplying the estimated OSA prevalence in the provincial PSG cohort by the proportion of the Ontario general population who underwent a PSG. Previous research suggests that up to 80% of individuals with moderate-severe OSA cases remain undiagnosed [24]. Accordingly, assuming that only 20% of true cases are captured in HAD, we derived a scenario-based upper-bound (projected maximum) estimate by scaling the observed prevalence by the inverse of this proportion (i.e. dividing by 0.20).

All statistical analyses were performed in the secure environment at ICES Ottawa following Ontario privacy standards using SAS Enterprise Guide 7.15 (SAS Institute Inc., Cary, NC).

## Results

The measurement cohort comprised 18 581 individuals (Table 1), with a mean (SD) age of 50 years (14); 42.1% were female, and 14% resided in rural areas. The most prevalent comorbidities were hypertension (39.6%) and diabetes (17.7%). In the measurement cohort, 41.3% had moderate-severe and 21.6% had severe OSA. The provincial PSG cohort included 834 361 adults and resembled the measurement cohort’s baseline characteristics.

**Table 1:** Baseline characteristics of the study cohorts.

Variables	Value	Measurement	Population	ASD
		*N* = 18 581	*N* = 834 361	
Age	Mean ± SD	50.44 ± 14.25	50.41 ± 14.55	0
Age group	18–25	869 (4.7%)	38 749 (4.6%)	0
	26–40	3721 (20.0%)	177 334 (21.3%)	0.03
	41–54	6623 (35.6%)	288 505 (34.6%)	0.02
	55–64	4295 (23.1%)	183 704 (22.0%)	0.03
	65+	3073 (16.5%)	146 069 (17.5%)	0.03
Sex	Female	7815 (42.1%)	359 658 (43.1%)	0.02
	Male	10 766 (57.9%)	474 703 (56.9%)	0.02
Income quintile (Q)	Q1	3036 (16.3%)	151 347 (18.1%)	0.05
	Q2	3569 (19.2%)	164 211 (19.7%)	0.01
	Q3	3670 (19.8%)	168 723 (20.2%)	0.01
	Q4	4103 (22.1%)	174 663 (20.9%)	0.03
	Q5	4125 (22.2%)	171 850 (20.6%)	0.04
Rural		2597 (14.0%)	89 443 (10.7%)	0.10
Charlson Comorbidity		2256 (12.1%)	90 414 (10.8%)	0.04
COPD		2216 (11.9%)	93 653 (11.2%)	0.02
Diabetes		3297 (17.7%)	139 311 (16.7%)	0.03
Hypertension		7355 (39.6%)	328 177 (39.3%)	0.01
CHF		858 (4.6%)	32 094 (3.8%)	0.04
Arrhythmia		497 (2.7%)	20 859 (2.5%)	0.01
Stroke		187 (1.0%)	8508 (1.0%)	0
Probability moderate-severe OSA	Mean ± SD	0.39 ± 0.26	0.44 ± 0.28	0.19
Probability severe OSA	Mean ± SD	0.13 ± 0.14	0.18 ± 0.17	0.27

ASD, absolute standardized difference; CHF, congestive heart failure; COPD, chronic obstructive pulmonary disease.

Based on the case-ascertainment algorithm, the mean (SD) predicted probability of moderate-severe OSA for the measurement and provincial PSG cohorts was 39% (26%) and 44% (28%), respectively. The mean (SD) predicted probability of severe OSA for the measurement and provincial PSG cohorts was 13% (18%) and 18% (17%). The histograms and density plots show empirical distributions (Supplementary Fig. S1).

### Measurement cohort: moderate-severe OSA

The Youden Index yielded a probability threshold of >38%, which equaled the optimal prevalence threshold (sensitivity: 70%, specificity: 80%). The optimal RR threshold was >26% (sensitivity: 83%, specificity: 62%).

**Figure 1:**
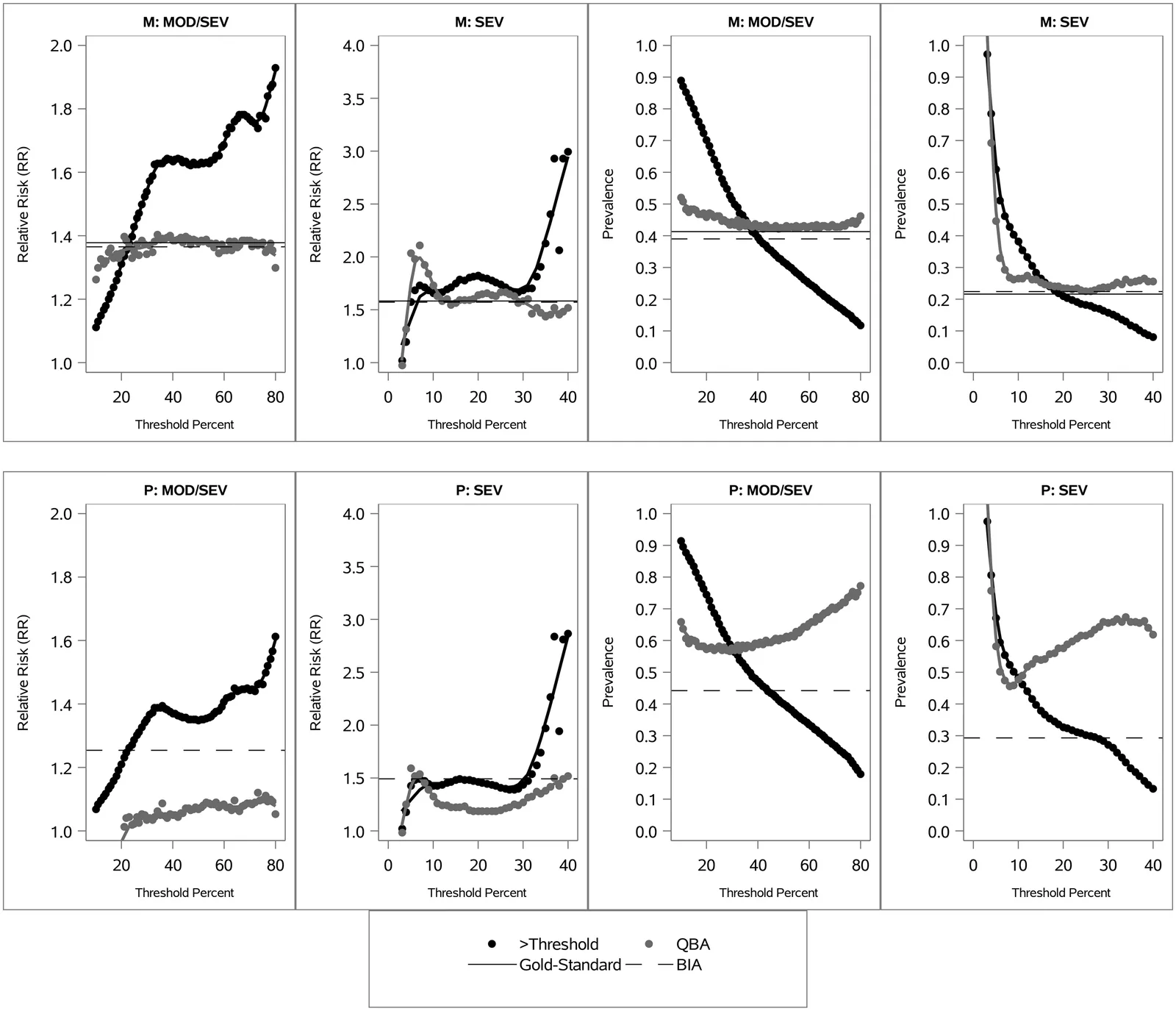
The figures compare estimates across the alternative definitions for moderate to severe (MOD/SEV) and severe OSA (SEV) Row 1 is for the Measurement (M) cohort and the row 2 is for the Provincial (P) cohort. The covariate mean RR and prevalence are graphed on the *y*-axis. The *x*-axis shows the threshold probability used to define surrogate case status. Across all plots, the black dotted line represents ach threshold probability, and the gray dotted lines is the threshold-specific QBA correction. The horizontal solid black line is gold-standard estimate, whereas the dashed black line is the BIA estimates.

We compared each OSA surrogate case definition against the gold-standard definition of moderate-severe OSA. Across all thresholds, the >38% threshold provided the lowest relative bias when averaged across covariate prevalences (Fig. 1). Overall, across covariate prevalences, the mean relative bias for >38%, >26%, and BIA was 8.2%, 48.2%, and 5.3%, respectively. Thus, the BIA surrogate had the lowest relative bias compared to the gold standard. For example, among individuals >50 years old, the gold-standard, >38%, >26%, and BIA prevalence were 49.4%, 55.6%, 87.3%, and 47.8%, respectively (Table 2).

**Table 2:** Overall and covariate-specific estimates by surrogate for moderate-severe OSA in the measurement cohort with relative bias estimates compared to the gold standard.

	Gold-standard	**>38% optimal prevalence threshold** ^a^	>26% optimal RR threshold	BIA
Prevalence (relative bias)				
Overall	0.413	0.413 (0)	0.579 (40.2)	0.383 (5.3)
Male	0.495	0.516 (4.2)	0.772 (56)	0.448 (7.7)
Female	0.300	0.273 (9)	0.458 (52.7)	0.289 (0.3)
Age > 50	0.494	0.556 (12.6)	0.873 (76.7)	0.468 (3.2)
Age ≤ 50	0.329	0.266 (19.1)	0.569 (72.9)	0.291 (8.5)
Income—high	0.405	0.41 (1.2)	0.34 (16)	0.383 (3)
Income—low	0.419	0.416 (0.7)	0.351 (16.2)	0.379 (7.4)
Rural—Y	0.442	0.461 (4.3)	0.666 (50.7)	0.406 (3.8)
Rural—N	0.408	0.406 (0.5)	0.614 (50.5)	0.377 (5.6)
Charlson—Y	0.559	0.649 (16.1)	0.81 (44.9)	0.499 (7)
Charlson—N	0.393	0.381 (3.1)	0.571 (45.3)	0.365 (5.1)
COPD—Y	0.486	0.565 (16.3)	0.877 (80.5)	0.455 (2.3)
COPD—N	0.403	0.393 (2.5)	0.723 (79.4)	0.372 (6)
Hypertension—Y	0.530	0.576 (8.7)	0.745 (40.6)	0.473 (8.7)
Hypertension—N	0.336	0.307 (8.6)	0.471 (40.2)	0.321 (1.8)
Diabetes—Y	0.539	0.587 (8.9)	0.69 (28)	0.471 (9.5)
Diabetes—N	0.386	0.376 (2.6)	0.492 (27.5)	0.362 (4.1)
CHF—Y	0.607	0.713 (17.5)	0.875 (44.2)	0.519 (9.2)
CHF—N	0.403	0.399 (1)	0.587 (45.7)	0.375 (5)
Arrhythmia—Y	0.610	0.753 (23.4)	0.883 (44.8)	0.523 (6.9)
Arrhythmia—N	0.407	0.404 (0.7)	0.593 (45.7)	0.378 (5.2)
Stroke—Y	0.567	0.717 (26.5)	0.882 (55.6)	0.485 (1.8)
Stroke—N	0.411	0.41 (0.2)	0.637 (55)	0.382 (5.4)
RR (relative bias)^b^				
Male	1.651	1.892 (14.6)	1.685 (2.1)	1.471 (7.2)
Age > 50	1.505	2.086 (38.6)	1.535 (2)	1.531 (5.5)
Income—high	0.969	0.984 (1.5)	0.969 (0)	0.977 (4.4)
Rural—Y	1.084	1.135 (4.7)	1.084 (0)	1.051 (1.8)
Charlson—Y	1.424	1.704 (19.7)	1.418 (0.4)	1.334 (2.1)
COPD—Y	1.208	1.437 (19)	1.212 (0.3)	1.19 (3.8)
Hypertension—Y	1.579	1.876 (18.8)	1.583 (0.3)	1.419 (7)
Diabetes—Y	1.397	1.56 (11.7)	1.403 (0.4)	1.269 (5.5)
CHF—Y	1.506	1.788 (18.7)	1.491 (1)	1.353 (4.4)
Arrhythmia—Y	1.497	1.862 (24.4)	1.49 (0.5)	1.354 (1.7)
Stroke—Y	1.379	1.746 (26.6)	1.384 (0.4)	1.245 (3.9)

CHF, congestive heart failure; COPD, chronic obstructive pulmonary disease.

a Equivalent to the Youden Index.

b Relative risks are compared between binary covariate values, e.g. male vs female, yes (Y) versus no (N).

In contrast, the >26% threshold had the lowest relative bias (Table 2, Fig. 1) for the covariate RR estimates. The covariate RR mean relative bias for >38%, >26%, and BIA was 18%, 0.7%, and 4.3%, respectively. Thus, the >26% surrogate had the lowest relative bias compared to the gold standard. For example, comparing individuals aged > 50 to ≤50 years old, the gold-standard, >38%, >26%, and BIA RRs were 1.51, 2.09, 1.53, and 1.59, respectively.

### Measurement cohort: severe OSA

Since the severe OSA prediction model had worse external calibration, the Youden Index yielded a probability threshold of >8%, which diverged from the optimal prevalence threshold of >19% (sensitivity: 49%, specificity: 87%). The RR optimal threshold was >12% (sensitivity: 67%, specificity: 79%).

We compared the OSA surrogate case definitions against the gold-standard definition of severe OSA. Across all thresholds, the >19% threshold provided the lowest relative bias when averaged across covariate prevalences (Fig. 1). Overall, across covariate prevalences, the mean relative bias for >19%, >12%, and BIA was 100%, 102%, and 47.5%, respectively. Thus, the BIA surrogate had the lowest relative bias compared to the gold standard. For example, for individuals >50 years, the gold-standard, >19%, >12%, and BIA prevalence was 26.3%, 48.7%, 56.6%, and 15.3%, respectively (Table 3).

**Table 3:** Overall and covariate-specific estimates by surrogate for severe OSA in the measurement cohort with relative bias estimates compared to the gold standard.

	Gold standard	**>19% optimal prevalence threshold** ^a^	>12% optimal RR threshold	BIA
Prevalence (relative bias)				
Overall	0.216	0.219 (1.4)	0.305 (41.2)	0.218 (3.7)
Male	0.275	0.476 (73.1)	0.428 (55.6)	0.194 (26.2)
Female	0.135	0.364 (169.6)	0.314 (132.6)	0.231 (77)
Age > 50	0.263	0.566 (115.2)	0.487 (85.2)	0.146 (41.8)
Age ≤ 50	0.168	0.287 (70.8)	0.27 (60.7)	0.283 (73.8)
Income—high	0.206	0.413 (100.5)	0.445 (116)	0.218 (9.7)
Income—low	0.224	0.441 (96.9)	0.477 (112.9)	0.213 (0.9)
Rural—Y	0.248	0.469 (89.1)	0.275 (10.9)	0.214 (11.3)
Rural—N	0.211	0.422 (100)	0.233 (10.4)	0.23 (16.6)
Charlson—Y	0.340	0.763 (124.4)	0.938 (175.9)	0.198 (40)
Charlson—N	0.199	0.382 (92)	0.565 (183.9)	0.343 (83.4)
COPD—Y	0.269	0.608 (126)	0.967 (259.5)	0.208 (20.4)
COPD—N	0.209	0.404 (93.3)	0.759 (263.2)	0.28 (43.1)
Hypertension—Y	0.302	0.604 (100)	0.222 (26.5)	0.168 (42.4)
Hypertension—N	0.159	0.314 (97.5)	0.117 (26.4)	0.288 (88.1)
Diabetes—Y	0.325	0.813 (150.2)	0.998 (207.1)	0.184 (41.5)
Diabetes—N	0.192	0.346 (80.2)	0.526 (174)	0.365 (99)
CHF—Y	0.394	0.815 (106.9)	0.247 (37.3)	0.211 (44.9)
CHF—N	0.207	0.41 (98.1)	0.131 (36.7)	0.344 (83.1)
Arrhythmia—Y	0.388	0.769 (98.2)	0.769 (98.2)	0.214 (43.3)
Arrhythmia—N	0.211	0.419 (98.6)	0.419 (98.6)	0.33 (76.8)
Stroke—Y	0.374	0.802 (114.4)	0.626 (67.4)	0.216 (40.6)
Stroke—N	0.214	0.425 (98.6)	0.329 (53.7)	0.328 (85.5)
RR (relative bias)^b^				
Male	2.040	1.307 (35.9)	1.365 (33.1)	1.112 (42.4)
Age > 50	1.568	1.975 (26)	1.802 (14.9)	1.801 (21.6)
Income—high	0.923	0.937 (1.5)	0.934 (1.2)	0.933 (6.6)
Rural—Y	1.177	1.111 (5.6)	1.176 (0.1)	1.035 (4.9)
Charlson—Y	1.713	1.995 (16.5)	1.66 (3.1)	1.664 (4.3)
COPD—Y	1.288	1.505 (16.8)	1.275 (1)	1.305 (8.6)
Hypertension—Y	1.897	1.925 (1.5)	1.892 (0.3)	1.627 (9.5)
Diabetes—Y	1.686	2.35 (39.4)	1.896 (12.5)	1.906 (19.5)
CHF—Y	1.900	1.988 (4.6)	1.893 (0.4)	1.582 (7.9)
Arrhythmia—Y	1.839	1.833 (0.3)	1.833 (0.3)	1.49 (7.8)
Stroke—Y	1.747	1.888 (8.1)	1.903 (8.9)	1.469 (2.1)

CHF, congestive heart failure; COPD, chronic obstructive pulmonary disease.

a Not equivalent to the Youden Index.

b Relative risks are compared between binary covariate values, e.g. male vs female, yes (Y) versus no (N).

**Figure 2:**
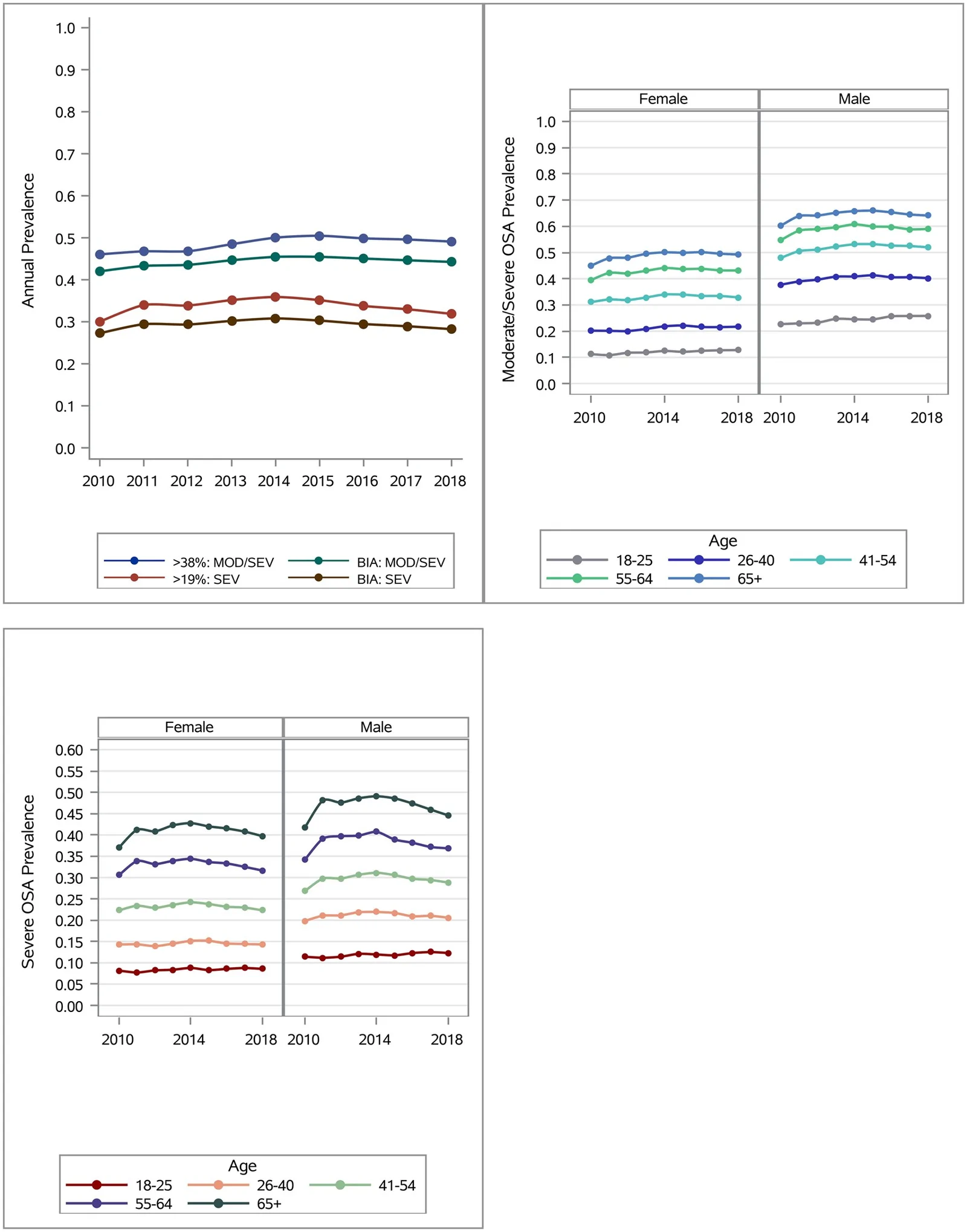
Annual point prevalence across alternative definitions and by age and sex for the BIA definition only.

In contrast, the >12% threshold had the lowest relative bias when averaged across covariate RRs (Table 3, Fig. 2). The covariate RR mean relative bias for >19%, >12%, and BIA was 14.2%, 6.9%, and 12.3%, respectively. Thus, the >12% surrogate had the lowest relative bias compared to the gold standard. For example, comparing individuals aged >50 to ≤50 years, the gold-standard, >19%, >12%, and BIA RR were 1.57, 1.98, 1.80, and 1.91, respectively.

### Provincial PSG cohort

We applied three surrogate case definitions to the provincial PSG cohort and applied the simple QBA correction. However, the simple QBA overestimated the average prevalences and underestimated average RRs (Fig. 1). The individual covariate prevalence and RR values can be found in the data supplement (Supplementary Figs S5 and S6).

Among adult Ontarians who underwent diagnostic PSG, the >38% threshold and BIA estimate the prevalence at 48.6% and 44.2% for moderate-severe OSA and at 33.1% and 29.3% for severe OSA (Table 4). Moderate-severe OSA prevalence was highest for men ≥65 years old, being considerably higher using the >38% threshold (87.8%) compared to BIA (64.2%). A similar pattern was observed for severe OSA. The annual prevalence for moderate-severe and severe OSA was stable annually across all definitions and sociodemographic sub-groups (Fig. 2).

**Table 4:** Prevalence measures in the population cohort for those referred to overnight PSG.

Strata	>38%-moderate-severe OSA	BIA-moderate-severe OSA	>19%-severe OSA	**BIA-severe OSA**
Overall	0.486 (0.485–0.487)	0.442 (0.442–0.443)	0.335 (0.334–0.336)	0.293 (0.292–0.294)
18–25	0.151 (0.147–0.154)	0.186 (0.183–0.19)	0.094 (0.091–0.097)	0.103 (0.1–0.106)
26–40	0.332 (0.33–0.334)	0.328 (0.326–0.33)	0.225 (0.223–0.227)	0.186 (0.183–0.187)
41–54	0.444 (0.442–0.446)	0.435 (0.434–0.437)	0.311 (0.309–0.312)	0.268 (0.266–0.27)
55–64	0.597 (0.595–0.599)	0.513 (0.51–0.515)	0.406 (0.404–0.408)	0.357 (0.354–0.359)
65+	0.704 (0.702–0.707)	0.575 (0.572–0.577)	0.493 (0.49–0.495)	0.441 (0.438–0.444)
Female	0.347 (0.345–0.349)	0.348 (0.346–0.349)	0.281 (0.28–0.283)	0.263 (0.261–0.264)
Male	0.591 (0.59–0.593)	0.514 (0.513–0.516)	0.376 (0.375–0.378)	0.315 (0.314–0.317)
Female: 18–25	0.046 (0.043–0.049)	0.121 (0.116–0.126)	0.054 (0.051–0.058)	0.085 (0.08–0.088)
Female: 26–40	0.175 (0.172–0.178)	0.212 (0.209–0.214)	0.144 (0.142–0.147)	0.145 (0.142–0.148)
Female: 41–54	0.352 (0.35–0.355)	0.328 (0.326–0.331)	0.247 (0.245–0.25)	0.231 (0.229–0.234)
Female: 55–64–	0.438 (0.435–0.442)	0.427 (0.423–0.43)	0.363 (0.36–0.366)	0.329 (0.326–0.332)
Female: 65+	0.483 (0.479–0.487)	0.489 (0.485–0.492)	0.447 (0.443–0.451)	0.409 (0.405–0.413)
Male: 18–25	0.244 (0.238–0.25)	0.245 (0.24–0.251)	0.129 (0.125–0.134)	0.119 (0.115–0.124)
Male: 26–40	0.430 (0.427–0.433)	0.401 (0.398–0.403)	0.275 (0.272–0.278)	0.211 (0.208–0.213)
Male: 41–54	0.513 (0.511–0.516)	0.516 (0.513–0.518)	0.358 (0.356–0.361)	0.295 (0.293–0.297)
Male: 55–64–	0.735 (0.732–0.737)	0.587 (0.584–0.591)	0.443 (0.44–0.447)	0.381 (0.378–0.384)
Male: 65+	0.878 (0.876–0.881)	0.643 (0.639–0.646)	0.528 (0.525–0.532)	0.467 (0.463–0.471)

The BIA surrogate applied to the provincial PSG cohort yielded similar crude covariate RRs as observed in the measurement cohort for moderate-severe OSA and severe OSA. Higher crude RRs were observed for age, males, higher-income quintiles, rurality, comorbidities and over time (Table 5). Similar patterns were observed using threshold-based definitions (Table 5).

**Table 5:** Crude RR of OSA in the population cohort using the BIA and optimal RR threshold surrogates.

	Moderate-severe OSA		Severe OSA	
	BIA RR (95% CI)	>26% RR (95% CI)	BIA RR (95% CI)	>12% RR (95% CI)
Age (per 5 years)	1.083 (1.082–1.083)	1.106 (1.106–1.107)	1.123 (1.122–1.124)	1.11 (1.109–1.111)
Male	1.478 (1.471–1.486)	1.623 (1.618–1.629)	1.202 (1.193–1.209)	1.299 (1.293–1.306)
Income Q1	0.968 (0.96–0.974)	0.955 (0.95–0.96)	1.033 (1.022–1.045)	1.047 (1.039–1.055)
Income Q2	0.989 (0.982–0.995)	0.972 (0.967–0.977)	1.044 (1.032–1.056)	1.05 (1.042–1.058)
Income Q3	0.989 (0.981–0.996)	0.975 (0.971–0.98)	1.028 (1.016–1.039)	1.037 (1.029–1.045)
Income Q4	0.999 (0.993–1.007)	0.989 (0.984–0.994)	1.02 (1.009–1.031)	1.026 (1.018–1.034)
Rurality	1.04 (1.031–1.046)	1.029 (1.024–1.035)	1.119 (1.107–1.131)	1.149 (1.141–1.158)
Charlson	1.225 (1.217–1.233)	1.266 (1.26–1.272)	1.62 (1.607–1.633)	1.518 (1.508–1.528)
Hypertension	1.34 (1.334–1.346)	1.407 (1.402–1.411)	1.621 (1.61–1.632)	1.561 (1.554–1.569)
CHF	1.282 (1.273–1.294)	1.343 (1.334–1.353)	1.634 (1.611–1.652)	1.623 (1.606–1.639)
COPD	1.177 (1.167–1.185)	1.229 (1.223–1.235)	1.324 (1.311–1.335)	1.299 (1.29–1.308)
Arrhythmia	1.269 (1.253–1.281)	1.317 (1.305–1.33)	1.555 (1.53–1.577)	1.538 (1.518–1.557)
Stroke	1.203 (1.18–1.227)	1.247 (1.229–1.265)	1.608 (1.575–1.643)	1.573 (1.543–1.604)
Annual slope	1.006 (1.005–1.007)	1.007 (1.006–1.008)	1.002 (1.001–1.003)	1.005 (1.004–1.006)

CHF, congestive heart failure; COPD, chronic obstructive pulmonary disease.

Using the 2016 Canadian Census of Ontario as the general population denominator (*N* = 11 040 866), 7.7% of the general population underwent a PSG from 2010 to 2018, which provides a lower general population scenario-based projected prevalence minimum of 3.4% for moderate-severe OSA using the BIA method. Assuming that up to 80% of cases of moderate-severe OSA remain undiagnosed yields a scenario-based projected prevalence maximum of 17.1%. The minimum and maximum moderate-severe OSA scenario-based projections for females and males were 2.2% to 11.2% and 4.7% to 23.5%, respectively. For those aged 18–25, 26–40, 41–54, 55–64, and 65+, the lower and upper prevalence limits were 0.5%–2%, 1.5%–7.5%, 4.7%–23.3%, 5.1%–25.6%, and 3.7%–18.6%, respectively.

## Discussion

This population-based study of the most populous province in Canada (>10 000 000 adults) updates OSA epidemiology using a probabilistic HAD-based algorithm, combined with misclassification adjustment. We estimate that in Ontario, 44.2% of adults who underwent a diagnostic PSG have moderate-severe OSA and 29.3% have severe OSA. This projects a general population prevalence of 3.4% for moderate-severe OSA, which, depending on the undiagnosed OSA rate, may be as high as 17.1%. Our methodology also reproduced well-established OSA correlates, such as advanced age, male sex, and hypertension [25–27] and demonstrated a stable annual trend.

We demonstrate the challenges of using a single threshold probability (e.g. >50%) when defining disease status, as different thresholds can substantially affect estimates of prevalence and associations, and no single threshold is optimal across all study objectives. We identify probability thresholds of >38% for estimating moderate-severe OSA prevalence and >26% for RR factors as reasonable choices. The former was determined using the Youden Index, and the latter was derived from the measurement cohort to minimize the average relative bias across included covariates compared to the gold standard. In contrast, the Youden Index was not optimal for prevalence estimation for severe OSA, likely due to poorer model calibration. For severe OSA, we identify a >19% prevalence threshold and a >12% RR threshold, albeit with lower accuracy than for moderate-severe OSA.

The simple QBA approach, which corrects summary-level statistics using external covariate-specific sensitivity and specificity estimates, should be used with caution. Its performance declined when applied outside the measurement cohort, limiting generalizability. In contrast, the BIA produced the most accurate (i.e. lowest relative bias) and generalizable estimates. Unlike threshold-based approaches, BIA is agnostic to single probability cutoffs, instead averaging across possible thresholds and, thereby, maximizing the use of available information. Previous applications of BIA relied on highly discriminative models for conditions such as Colles Fracture (c-statistic > 0.95) [12, 13]. OSA, however, represents a heterogeneous disease, with overlapping symptoms, reliance on PSG for diagnosis, and inconsistent coding in HAD, resulting in models with moderate discrimination and greater vulnerability to misclassification. Despite these challenges, the BIA method yielded more accurate prevalence and RRs than single-threshold methods relative to the gold standard. To our knowledge, this is the first application of the BIA approach to a probability model with moderate discrimination.

Population-based studies are essential for estimating the population burden of OSA, despite well-recognized challenges in applying OSA diagnostic criteria at scale. Prior global estimates have relied on extrapolation from clinic-based samples, which may overestimate prevalence due to referral bias [28]. Using misclassification-adjusted methods applied to population-level data, we estimated a general population OSA scenario-based projected prevalence of 3.4%–17.1%, dependent on assumptions regarding underdiagnosis. These estimates are broadly consistent with a province-wide study in Alberta, Canada (3.5%–15.2%) [29], and with the global estimate of approximately 13% for moderate-severe OSA in adults aged 30–69 years [28]. Our findings reproduce well-established epidemiologic patterns of OSA, including higher prevalence among men, older adults, and individuals with hypertension [25–27]. We also observed temporal stability, supporting the internal validity of probabilistic approaches.

This study also provides methodological guidance by demonstrating the use of probabilistic case ascertainment models as surrogate disease definitions in population-based research. Although HAD offers near-complete population coverage, its validity depends on how well surrogate definitions reflect true disease status. We therefore advocate for probabilistic case-ascertainment algorithms rather than single deterministic definitions. In OSA, we show that probability-based surrogates can recover key epidemiologic measures despite imperfect ascertainment. Accuracy and generalizability were further improved using threshold-agnostic bootstrap imputation, which directly propagates uncertainty across the full range of threshold probabilities.

We acknowledge several limitations. Generalizability of the case-ascertainment models is restricted to individuals with a sufficient pre-test probability to be referred for and complete diagnostic PSG. While we can accurately estimate the prevalence within the population undergoing PSG, general population estimates rely on scenario-based projections with strong assumptions about population underdiagnosis. These estimates are subject to additional variability beyond the scope of what the BIA method can account for. Furthermore, poorer calibration of the severe OSA model likely contributed to the higher relative biases observed for severe OSA estimates, warranting more cautious interpretation [10]. Future research should evaluate what degree of miscalibration offsets the observed misclassification mitigation when using BIA. We evaluated only select QBA methods. Other methods, particularly simulation-based or missing-data frameworks, could also be applied to address misclassification [30]. Accordingly, future studies should evaluate whether incorporating uncertainty into predicted probability estimation, by sampling probability distributions rather than assigning fixed predicted probabilities, further reduces misclassification bias. Furthermore, the selected probability thresholds were not optimism corrected or externally validated. This conveys another benefit of the BIA approach, as the choice of threshold probability is unnecessary. The probability model was derived, validated, and evaluated within a single province operating under a publicly funded healthcare system, and performance may differ in other settings. Nonetheless, our approach provides a reproducible template that can be adapted and re-evaluated in other jurisdictions to support accurate population-level OSA case ascertainment.

## Conclusion

Population-based probabilistic OSA case ascertainment using HAD enables robust estimation of OSA epidemiology, including prevalence and associations with key sociodemographic and clinical factors. Reliable application requires careful evaluation of probability thresholds and, where feasible, threshold-agnostic adjustment for misclassification, such as bootstrap imputation. Using this framework, we estimate that 44.2% of individuals referred for sleep assessment have moderate-severe OSA, corresponding to a general population scenario-based projected prevalence of 3.4%–17.1%. These methods provide a generalized framework for reducing misclassification and strengthening population-based inference using routinely collected health data.

## Ethics approval

ICES is a prescribed entity under Ontario’s Personal Health Information Protection Act (PHIPA). Section 45 of PHIPA authorizes ICES to collect personal health information, without consent, for the purpose of analysis or compiling statistical information with respect to the management of, evaluation or monitoring of the allocation of resources to or planning for all or part of the health system. Projects that use data collected by ICES under Section 45 of PHIPA, and use no other data, are exempt from REB review. The use of the data in this project is authorized under Section 45 and approved by ICES’ Privacy and Legal Office. All methods were carried out in accordance with relevant guidelines and regulations. The study datasets were linked using unique encoded identifiers and analyzed in the secure environment at ICES following Ontario privacy standards. Authors had no access to information that could identify individual participants during or after data collection.

## Supplementary Material

dyag176_Supplementary_Data

## Data Availability

Data may be obtained from a third party and are not publicly available. In Ontario, the dataset from this study is held securely in coded form at ICES. While data sharing agreements prohibit ICES from making the dataset publicly available, access may be granted to those who meet prespecified criteria for confidential access, available at www.ices.on.ca/DAS (email: das@ices.on.ca). The full dataset creation plan and underlying analytical code are available from the authors upon request, understanding that the computer programs may rely upon coding templates or macros that are unique to ICES and are therefore either inaccessible or may require modification.
