# Supplementary material for: A population-based study of obstructive sleep apnea: using probabilistic case ascertainment through health administrative databases
Source: Int J Epidemiol. 2026 Aug 20;55(5):dyag176. doi: 10.1093/ije/dyag176 (PMC13493051; doi:10.1093/ije/dyag176)
Supplement: Supplementary file 1 [file dyag176_supplementary_data.zip › ije-2026-01-0177-File006.pdf]

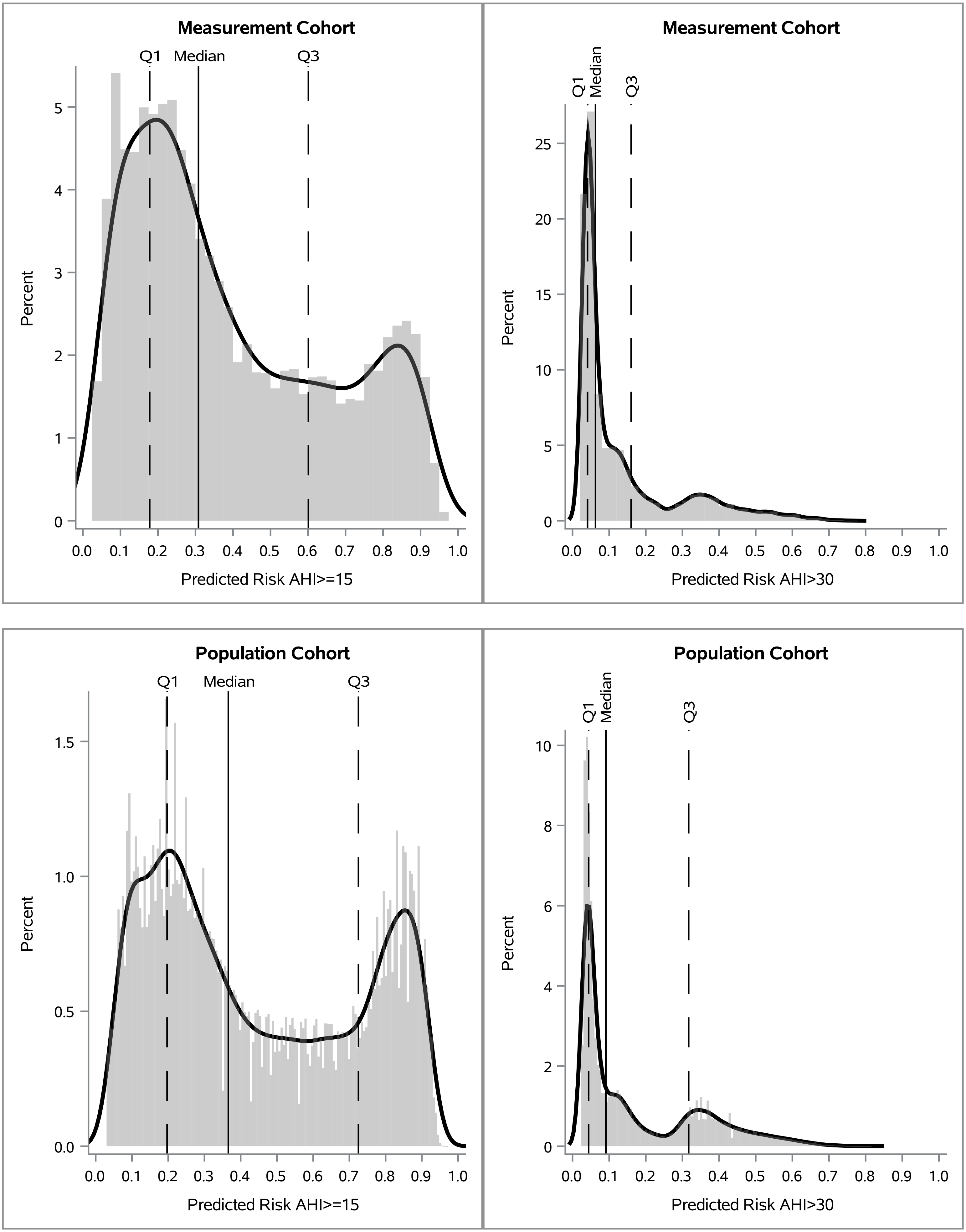

Measurement Cohort
Q1
Median
Q3
Percent
Predicted Risk AHI>=15
Measurement Cohort
Q1
Median
Q3
Percent
Predicted Risk AHI>30
Population Cohort
Q1
Median
Q3
Percent
Predicted Risk AHI>=15
Population Cohort
Q1
Median
Q3
Percent
Predicted Risk AHI>30
