# Supplementary material for: A population-based study of obstructive sleep apnea: using probabilistic case ascertainment through health administrative databases
Source: Int J Epidemiol. 2026 Aug 20;55(5):dyag176. doi: 10.1093/ije/dyag176 (PMC13493051; doi:10.1093/ije/dyag176)
Supplement: Supplementary file 1 [file dyag176_supplementary_data.zip › ije-2026-01-0177-File005.docx]

Data Supplement

**Title:** A Population-Based Study of Obstructive Sleep Apnea: Using Probabilistic Case Ascertainment through Health Administrative Databases

**Authors**: Robert Talarico, Marcus Povitz, Sachin R. Pendharkar, William Reisman, Daniel I. McIsaac, Tetyana Kendzerska

Table S1: Details on databases utilized in the current study.

Table S2: Surrogate obstructive sleep apnea (OSA) definitions.

Table S3: Operating characteristics for every threshold for moderate-severe obstructive sleep apnea (OSA) model.

Table S4: Operating characteristics for every threshold for severe obstructive sleep apnea (OSA) model.

Table S5: Covariate specific operating characteristics for simple quantitative bias analysis (QBA) analysis (moderate to severe obstructive sleep apnea (OSA)).

Table S6: Covariate specific operating characteristics for simple quantitative bias analysis (QBA) analysis (severe obstructive sleep apnea (OSA)).

Table S7: STROBE/RECORD Reporting Checklist

Table S8: Bootstrap Imputation Approach (BIA) Schematic Diagram and Short Summary Table of Prior Studies

Figure S1: Histogram with overlaid density plots of Model-Based predicted probabilities across the measurement and population cohort for moderate-severe and severe obstructive sleep apnea (OSA). The solid black line displays the median predicted probability and the dashed lines the 25^th^ and 7^5th^ percentiles. The y-axis shows the percent of the cohort falling into each bin and the x-axis is the model-based predicted probability ranging from 0 to 1.

Figure S2: Individual Covariate RR bias plots (akin to Figure 2). The legend indicates the labelling and the BIA approach is named, ‘Probabilistic’ , the Gold-Standard ‘Reference’, and ‘Bias-corrected’ is the threshold simple QBA approach

Figure S3: Individual Prevalence bias plots (akin to Figure 2) for the opposite level of Figure S4. The legend indicates the labels and the BIA approach is named, ‘Probabilistic’, the Gold-Standard ‘Reference’, and ‘Bias-corrected’ is the threshold simple QBA approach

Figure S4: Individual Prevalence bias pots (akin to Figure 2) for the opposite level as Figure S3. The legend indicates the labels and the BIA approach is named, ‘Probabilistic’, the Gold-Standard ‘Reference’, and ‘Bias-corrected’ is the threshold simple QBA approach

S-References.

**Table S1: Details on databases utilized in the current study.**

**Clinical Data (Prior Model Development Cohorts)**

In a prior study, we developed and validated probabilistic obstructive sleep apnea (OSA) case-ascertainment models using two academic sleep-centre cohorts linked to health administrative data (HAD).[1] Model derivation and internal validation were performed using the Ottawa Hospital (TOH) Sleep Database ([NCT03834792](https://clinicaltrials.gov/ct2/show/NCT03834792)), which contains clinical and PSG data on all consecutive adults who underwent a diagnostic sleep study in the TOH Sleep Center between 2015 and 2017. External validation was conducted in the London Health Sciences Centre Sleep and Apnea Assessment Unit PSG database, which includes clinical and PSG data on all consecutive adults who underwent a diagnostic sleep study in the London Health Sciences Centre between 2007 and 2015. These cohorts are used in the present study solely as the measurement cohort to evaluate misclassification and compare surrogate definitions.

**Health Administrative Data**

Ontario provides universal health coverage and maintains comprehensive, linkable HAD at ICES (formerly known as the Institute for Clinical and Evaluative Sciences), an independent research institute. Databases used included the Registered Persons Database (demographics); Discharge Abstract Database (hospitalizations); the OHIP database (physician billing and technical fees for procedures including PSGs); the Canadian Census (neighbourhood socioeconomic details); ICES Physician Database (physician specialty). We also utilized ICES-derived disease-specific databases. Finally, for all insured Ontario residents diagnosed with OSA by a sleep physician registered with the Assistive Devices Program (ADP), partial public funding for positive airway pressure (PAP) systems is provided and recorded in the ADP database. The databases listed were linked using unique encoded identifiers. A detailed description is available at [datadictionary.ices.on.ca/Applications/DataDictionary/](https://datadictionary.ices.on.ca/Applications/DataDictionary/).

**Table S2: Surrogate obstructive sleep apnea (OSA) definitions.**

| Moderate to Severe OSA | | | |
| --- | --- | --- | --- |
|  | | | |
| Surrogate | Method | Description | Operating Characteristics |
| >38% | Youden Index^1^ | Threshold based: Maximizes sum of sensitivity and specificity | Sensitivity: 70%  Specificity: 80% |
| >26% | Optimal RR Threshold | Threshold-based: Minimizes the relative bias across all covariate RR | Sensitivity: 83%  Specificity: 62% |
| BIA | Bootstrap Imputation Approach | Threshold-free | N/A |
| Severe OSA | | | |
|  | | | |
| Surrogate | Method | Description | Operating Characteristics |
| >19% | Optimal Prevalence Threshold^2^ | Threshold-based: Minimizes the relative bias across all covariate prevalence | Sensitivity: 49%  Specificity: 87% |
| >12% | Optimal RR Threshold | Minimizes the relative bias across all covariate RR | Sensitivity: 67%  Specificity: 79% |
| BIA | Bootstrap Imputation Approach | Threshold-free | N/A |

^1^Youden Index equal to the optimal prevalence threshold

^2^ Youden Index was not used due to poor model calibration

**Table S3: Operating characteristics for every threshold for moderate-severe obstructive sleep apnea (OSA) model.**

| **Probability Threshold** | **Sensitivity** | **Specificity** | **Positive Predictive Value** | **Negative Predictive Value** | **Youden** | **Positive Likelihood Ratio** | **Negative Likelihood Ratio** |
| --- | --- | --- | --- | --- | --- | --- | --- |
| 2 | 1.0000 | .0002 | .41278 | 1.00000 | .0002 | 1.00 | .00 |
| 3 | .9996 | .0099 | .41505 | .97297 | .0095 | 1.01 | .04 |
| 4 | .9987 | .0245 | .41843 | .96390 | .0232 | 1.02 | .05 |
| 5 | .9978 | .0481 | .42419 | .96863 | .0459 | 1.05 | .05 |
| 6 | .9944 | .0739 | .43007 | .94935 | .0683 | 1.07 | .08 |
| 7 | .9914 | .1021 | .43693 | .94407 | .0935 | 1.10 | .08 |
| 8 | .9854 | .1382 | .44555 | .93086 | .1236 | 1.14 | .11 |
| 9 | .9803 | .1696 | .45347 | .92458 | .1499 | 1.18 | .12 |
| 10 | .9752 | .2008 | .46167 | .92020 | .1760 | 1.22 | .12 |
| 11 | .9675 | .2312 | .46935 | .91017 | .1987 | 1.26 | .14 |
| 12 | .9626 | .2534 | .47537 | .90596 | .2160 | 1.29 | .15 |
| 13 | .9575 | .2771 | .48211 | .90269 | .2346 | 1.32 | .15 |
| 14 | .9506 | .3045 | .48995 | .89762 | .2551 | 1.37 | .16 |
| 15 | .9431 | .3301 | .49735 | .89203 | .2732 | 1.41 | .17 |
| 16 | .9366 | .3623 | .50792 | .89052 | .2989 | 1.47 | .17 |
| 17 | .9283 | .3891 | .51643 | .88532 | .3174 | 1.52 | .18 |
| 18 | .9199 | .4147 | .52485 | .88052 | .3346 | 1.57 | .19 |
| 19 | .9109 | .4433 | .53487 | .87627 | .3542 | 1.64 | .20 |
| 20 | .9029 | .4689 | .54438 | .87291 | .3718 | 1.70 | .21 |
| 21 | .8920 | .4994 | .55600 | .86809 | .3914 | 1.78 | .22 |
| 22 | .8798 | .5271 | .56664 | .86185 | .4069 | 1.86 | .23 |
| 23 | .8696 | .5528 | .57745 | .85779 | .4224 | 1.94 | .24 |
| 24 | .8572 | .5795 | .58891 | .85239 | .4367 | 2.04 | .25 |
| 25 | .8422 | .6053 | .59994 | .84517 | .4475 | 2.13 | .26 |
| 26 | .8331 | .6237 | .60877 | .84170 | .4568 | 2.21 | .27 |
| 27 | .8193 | .6448 | .61847 | .83543 | .4641 | 2.31 | .28 |
| 28 | .8088 | .6609 | .62638 | .83107 | .4697 | 2.39 | .29 |
| 29 | .7966 | .6801 | .63635 | .82630 | .4767 | 2.49 | .30 |
| 30 | .7839 | .7002 | .64764 | .82179 | .4841 | 2.61 | .31 |
| 31 | .7752 | .7109 | .65330 | .81816 | .4861 | 2.68 | .32 |
| 32 | .7594 | .7304 | .66439 | .81202 | .4898 | 2.82 | .33 |
| 33 | .7522 | .7385 | .66910 | .80922 | .4907 | 2.88 | .34 |
| 34 | .7375 | .7537 | .67785 | .80336 | .4912 | 2.99 | .35 |
| 35 | .7273 | .7626 | .68283 | .79917 | .4899 | 3.06 | .36 |
| 36 | .7146 | .7750 | .69061 | .79438 | .4896 | 3.18 | .37 |
| 37 | .7053 | .7834 | .69587 | .79090 | .4887 | 3.26 | .38 |
| 38 | .6951 | .7971 | .70656 | .78815 | .4922 | 3.43 | .38 |
| 39 | .6827 | .8087 | .71501 | .78389 | .4914 | 3.57 | .39 |
| 40 | .6748 | .8158 | .72025 | .78115 | .4906 | 3.66 | .40 |
| 41 | .6644 | .8262 | .72869 | .77789 | .4906 | 3.82 | .41 |
| 42 | .6589 | .8314 | .73306 | .77618 | .4903 | 3.91 | .41 |
| 43 | .6483 | .8421 | .74264 | .77309 | .4904 | 4.11 | .42 |
| 44 | .6410 | .8482 | .74802 | .77076 | .4892 | 4.22 | .42 |
| 45 | .6342 | .8529 | .75189 | .76841 | .4871 | 4.31 | .43 |
| 46 | .6239 | .8603 | .75844 | .76499 | .4842 | 4.47 | .44 |
| 47 | .6132 | .8674 | .76472 | .76140 | .4806 | 4.62 | .45 |
| 48 | .6056 | .8718 | .76849 | .75873 | .4774 | 4.72 | .45 |
| 49 | .5968 | .8766 | .77275 | .75573 | .4734 | 4.84 | .46 |
| 50 | .5872 | .8810 | .77625 | .75227 | .4682 | 4.93 | .47 |
| 51 | .5754 | .8865 | .78079 | .74816 | .4619 | 5.07 | .48 |
| 52 | .5671 | .8901 | .78389 | .74526 | .4572 | 5.16 | .49 |
| 53 | .5580 | .8958 | .79007 | .74250 | .4538 | 5.36 | .49 |
| 54 | .5501 | .8999 | .79439 | .74002 | .4500 | 5.50 | .50 |
| 55 | .5334 | .9052 | .79824 | .73410 | .4386 | 5.63 | .52 |
| 56 | .5250 | .9084 | .80103 | .73124 | .4334 | 5.73 | .52 |
| 57 | .5134 | .9125 | .80478 | .72737 | .4259 | 5.87 | .53 |
| 58 | .4994 | .9154 | .80581 | .72237 | .4148 | 5.90 | .55 |
| 59 | .4895 | .9176 | .80679 | .71891 | .4071 | 5.94 | .56 |
| 60 | .4783 | .9209 | .80953 | .71523 | .3992 | 6.05 | .57 |
| 61 | .4658 | .9258 | .81515 | .71146 | .3916 | 6.28 | .58 |
| 62 | .4568 | .9280 | .81674 | .70851 | .3848 | 6.34 | .59 |
| 63 | .4413 | .9315 | .81897 | .70344 | .3728 | 6.44 | .60 |
| 64 | .4337 | .9343 | .82266 | .70127 | .3680 | 6.60 | .61 |
| 65 | .4184 | .9379 | .82557 | .69647 | .3563 | 6.74 | .62 |
| 66 | .4072 | .9412 | .82948 | .69317 | .3484 | 6.93 | .63 |
| 67 | .3978 | .9435 | .83179 | .69034 | .3413 | 7.04 | .64 |
| 68 | .3865 | .9464 | .83516 | .68700 | .3329 | 7.21 | .65 |
| 69 | .3745 | .9493 | .83854 | .68349 | .3238 | 7.39 | .66 |
| 70 | .3664 | .9517 | .84207 | .68125 | .3181 | 7.59 | .67 |
| 71 | .3549 | .9545 | .84560 | .67797 | .3094 | 7.80 | .68 |
| 72 | .3439 | .9574 | .85010 | .67491 | .3013 | 8.07 | .69 |
| 73 | .3325 | .9596 | .85256 | .67165 | .2921 | 8.23 | .70 |
| 74 | .3212 | .9617 | .85491 | .66841 | .2829 | 8.39 | .71 |
| 75 | .3067 | .9641 | .85714 | .66427 | .2708 | 8.54 | .72 |
| 76 | .2934 | .9670 | .86207 | .66070 | .2604 | 8.89 | .73 |
| 77 | .2760 | .9689 | .86197 | .65569 | .2449 | 8.87 | .75 |
| 78 | .2648 | .9715 | .86721 | .65281 | .2363 | 9.29 | .76 |
| 79 | .2484 | .9737 | .86907 | .64830 | .2221 | 9.44 | .77 |
| 80 | .2279 | .9759 | .86922 | .64267 | .2038 | 9.46 | .79 |
| 81 | .2131 | .9784 | .87380 | .63886 | .1915 | 9.87 | .80 |
| 82 | .1939 | .9803 | .87368 | .63375 | .1742 | 9.84 | .82 |
| 83 | .1764 | .9828 | .87800 | .62934 | .1592 | 10.26 | .84 |
| 84 | .1576 | .9849 | .87991 | .62457 | .1425 | 10.44 | .86 |
| 85 | .1378 | .9871 | .88230 | .61963 | .1249 | 10.68 | .87 |
| 86 | .1230 | .9896 | .89215 | .61618 | .1126 | 11.83 | .89 |
| 87 | .0966 | .9925 | .90036 | .60987 | .0891 | 12.88 | .91 |
| 88 | .0788 | .9946 | .91101 | .60570 | .0734 | 14.59 | .93 |
| 89 | .0601 | .9961 | .91468 | .60126 | .0562 | 15.41 | .94 |
| 90 | .0398 | .9977 | .92424 | .59652 | .0375 | 17.30 | .96 |
| 91 | .0236 | .9989 | .93782 | .59278 | .0225 | 21.45 | .98 |
| 92 | .0153 | .9997 | .97500 | .59092 | .0150 | 51.00 | .98 |
| 93 | .0065 | 1.0000 | 1.00000 | .58885 | .0065 | . | .99 |
| 94 | .0030 | 1.0000 | 1.00000 | .58799 | .0030 | . | 1.00 |
| 95 | .0007 | 1.0000 | 1.00000 | .58742 | .0007 | . | 1.00 |
| 96 | .0001 | 1.0000 | 1.00000 | .58730 | .0001 | . | 1.00 |
| 100 | .0000 | 1.0000 | . | .70280 | .0000 | . | 1.00 |

**Table S4: Operating characteristics for every threshold for severe obstructive sleep apnea (OSA) model.**

| **Probability Threshold** | **Sensitivity** | **Specificity** | **Positive Predictive Value** | **Negative Predictive Value** | **Youden** | **Positive Likelihood Ratio** | **Negative Likelihood Ratio** |
| --- | --- | --- | --- | --- | --- | --- | --- |
| .02 | .9960 | .0330 | .22097 | .96781 | .0290 | 1.03 | .12 |
| .03 | .9564 | .2592 | .26229 | .95572 | .2156 | 1.29 | .17 |
| .04 | .9045 | .4676 | .31875 | .94678 | .3721 | 1.70 | .20 |
| .05 | .8699 | .5781 | .36218 | .94164 | .4480 | 2.06 | .23 |
| .06 | .8457 | .6413 | .39367 | .93786 | .4870 | 2.36 | .24 |
| .07 | .8250 | .6793 | .41468 | .93377 | .5043 | 2.57 | .26 |
| .08 | .8086 | .7073 | .43201 | .93064 | .5159 | 2.76 | .27 |
| .09 | .7839 | .7306 | .44484 | .92468 | .5145 | 2.91 | .30 |
| .10 | .7500 | .7528 | .45515 | .91621 | .5028 | 3.03 | .33 |
| .11 | .7119 | .7729 | .46326 | .90689 | .4848 | 3.13 | .37 |
| .12 | .6660 | .7937 | .47067 | .89616 | .4597 | 3.23 | .42 |
| .13 | .6276 | .8091 | .47518 | .88752 | .4367 | 3.29 | .46 |
| .14 | .5935 | .8244 | .48208 | .88044 | .4179 | 3.38 | .49 |
| .15 | .5740 | .8375 | .49315 | .87715 | .4115 | 3.53 | .51 |
| .16 | .5523 | .8468 | .49820 | .87292 | .3991 | 3.61 | .53 |
| .17 | .5294 | .8544 | .50035 | .86830 | .3838 | 3.64 | .55 |
| .18 | .5127 | .8619 | .50553 | .86528 | .3746 | 3.71 | .57 |
| .19 | .4938 | .8678 | .50704 | .86159 | .3616 | 3.74 | .58 |
| .20 | .4835 | .8726 | .51106 | .85986 | .3561 | 3.80 | .59 |
| .21 | .4703 | .8779 | .51473 | .85753 | .3482 | 3.85 | .60 |
| .22 | .4566 | .8822 | .51635 | .85499 | .3388 | 3.88 | .62 |
| .23 | .4469 | .8871 | .52152 | .85346 | .3340 | 3.96 | .62 |
| .24 | .4364 | .8894 | .52082 | .85144 | .3258 | 3.95 | .63 |
| .25 | .4305 | .8914 | .52191 | .85038 | .3219 | 3.96 | .64 |
| .26 | .4225 | .8935 | .52202 | .84890 | .3160 | 3.97 | .65 |
| .27 | .4110 | .8966 | .52266 | .84682 | .3076 | 3.97 | .66 |
| .28 | .3991 | .9002 | .52406 | .84471 | .2993 | 4.00 | .67 |
| .29 | .3841 | .9034 | .52273 | .84194 | .2875 | 3.98 | .68 |
| .30 | .3696 | .9070 | .52255 | .83936 | .2766 | 3.97 | .70 |
| .31 | .3547 | .9125 | .52743 | .83700 | .2672 | 4.05 | .71 |
| .32 | .3350 | .9183 | .53039 | .83374 | .2533 | 4.10 | .72 |
| .33 | .3148 | .9230 | .52956 | .83027 | .2378 | 4.09 | .74 |
| .34 | .2909 | .9307 | .53631 | .82658 | .2216 | 4.20 | .76 |
| .35 | .2747 | .9357 | .54046 | .82408 | .2104 | 4.27 | .78 |
| .36 | .2552 | .9401 | .54008 | .82092 | .1953 | 4.26 | .79 |
| .37 | .2380 | .9456 | .54634 | .81839 | .1836 | 4.38 | .81 |
| .38 | .2186 | .9507 | .54984 | .81544 | .1693 | 4.43 | .82 |
| .39 | .2049 | .9554 | .55842 | .81355 | .1603 | 4.59 | .83 |
| .40 | .1902 | .9597 | .56519 | .81144 | .1499 | 4.72 | .84 |
| .41 | .1830 | .9616 | .56723 | .81038 | .1446 | 4.77 | .85 |
| .42 | .1687 | .9652 | .57179 | .80830 | .1339 | 4.85 | .86 |
| .43 | .1558 | .9681 | .57339 | .80636 | .1239 | 4.88 | .87 |
| .44 | .1486 | .9711 | .58604 | .80551 | .1197 | 5.14 | .88 |
| .45 | .1388 | .9732 | .58755 | .80406 | .1120 | 5.18 | .88 |
| .46 | .1306 | .9751 | .59076 | .80287 | .1057 | 5.24 | .89 |
| .47 | .1199 | .9774 | .59383 | .80131 | .0973 | 5.31 | .90 |
| .48 | .1074 | .9794 | .58960 | .79938 | .0868 | 5.21 | .91 |
| .49 | .1019 | .9810 | .59621 | .79866 | .0829 | 5.36 | .92 |
| .50 | .0940 | .9826 | .59841 | .79750 | .0766 | 5.40 | .92 |
| .51 | .0870 | .9848 | .61228 | .79662 | .0718 | 5.72 | .93 |
| .52 | .0815 | .9865 | .62405 | .79592 | .0680 | 6.04 | .93 |
| .53 | .0735 | .9884 | .63578 | .79483 | .0619 | 6.34 | .94 |
| .54 | .0646 | .9902 | .64428 | .79355 | .0548 | 6.59 | .94 |
| .55 | .0573 | .9916 | .65341 | .79253 | .0489 | 6.82 | .95 |
| .56 | .0526 | .9926 | .66144 | .79186 | .0452 | 7.11 | .95 |
| .57 | .0459 | .9933 | .65480 | .79082 | .0392 | 6.85 | .96 |
| .58 | .0409 | .9940 | .65339 | .79007 | .0349 | 6.82 | .96 |
| .59 | .0354 | .9952 | .66981 | .78932 | .0306 | 7.38 | .97 |
| .60 | .0294 | .9961 | .67429 | .78844 | .0255 | 7.54 | .97 |
| .61 | .0247 | .9968 | .67808 | .78774 | .0215 | 7.72 | .98 |
| .62 | .0192 | .9973 | .65812 | .78688 | .0165 | 7.11 | .98 |
| .63 | .0160 | .9976 | .64646 | .78639 | .0136 | 6.67 | .99 |
| .64 | .0137 | .9979 | .63953 | .78605 | .0116 | 6.52 | .99 |
| .65 | .0110 | .9984 | .65672 | .78568 | .0094 | 6.88 | .99 |
| .66 | .0087 | .9990 | .71429 | .78540 | .0077 | 8.70 | .99 |
| .67 | .0060 | .9992 | .68571 | .78497 | .0052 | 7.50 | .99 |
| .68 | .0040 | .9995 | .66667 | .78466 | .0035 | 8.00 | 1.00 |
| .69 | .0020 | .9995 | .53333 | .78434 | .0015 | 4.00 | 1.00 |
| .70 | .0017 | .9995 | .50000 | .78429 | .0012 | 3.40 | 1.00 |
| .71 | .0012 | .9996 | .45455 | .78422 | .0008 | 3.00 | 1.00 |
| .72 | .0007 | .9997 | .42857 | .78416 | .0004 | 2.33 | 1.00 |
| .73 | .0002 | .9998 | .25000 | .78409 | .0000 | 1.00 | 1.00 |
| .74 | .0002 | .9999 | .33333 | .78410 | .0001 | 2.00 | 1.00 |
| .76 | .0002 | 1.0000 | 1.00000 | .78412 | .0002 | . | 1.00 |
| 1.00 | .0000 | 1.0000 | . | .27538 | .0000 | . | 1.00 |

**Table S5: Covariate specific operating characteristics for simple quantitative bias analysis (QBA) analysis (moderate to severe obstructive sleep apnea (OSA)).**

| **Covariate** | **Threshold** | **Sensitivity (Covariate=0)** | **Sensitivity (Covariate=1)** | **Specificity (Covariate=0)** | **Specificity (Covariate=1)** |
| --- | --- | --- | --- | --- | --- |
| Age>50 | 38 | .5961 | .7589 | .8964 | .6690 |
|  | 26 | .7576 | .8819 | .7607 | .4474 |
| Arrythmia | 38 | .6875 | .8812 | .8029 | .4794 |
|  | 26 | .8281 | .9571 | .6302 | .2577 |
| Stroke | 38 | .6926 | .8774 | .7993 | .4938 |
|  | 26 | .8317 | .9434 | .6261 | .3086 |
| Charlson>0 | 38 | .6713 | .8160 | .8183 | .5859 |
|  | 26 | .8176 | .9128 | .6483 | .3789 |
| CHF | 38 | .6841 | .8464 | .8059 | .5223 |
|  | 26 | .8260 | .9328 | .6350 | .2700 |
| COPD | 38 | .6823 | .7746 | .8133 | .6582 |
|  | 26 | .8255 | .8794 | .6456 | .4359 |
| Diabetes | 38 | .6696 | .7798 | .8203 | .6542 |
|  | 26 | .8186 | .8812 | .6498 | .4629 |
| Hypertension | 38 | .6201 | .7685 | .8599 | .6616 |
|  | 26 | .7782 | .8862 | .7062 | .4457 |
| Income quintile: high | 38 | .6928 | .6981 | .7958 | .7987 |
|  | 26 | .8234 | .8456 | .6367 | .6077 |
| Male | 38 | .6289 | .7242 | .8847 | .7086 |
|  | 26 | .7310 | .8782 | .7966 | .4497 |
| Rural location | 38 | .6872 | .7430 | .8005 | .7750 |
|  | 26 | .8290 | .8580 | .6300 | .5749 |

**Table S6: Covariate specific operating characteristics for simple quantitative bias analysis (QBA) analysis (severe obstructive sleep apnea (OSA)).**

| **Covariate** | **Threshold** | **Sensitivity (Covariate=0)** | **Sensitivity (Covariate=1)** | **Specificity (Covariate=0)** | **Specificity (Covariate=1)** |
| --- | --- | --- | --- | --- | --- |
| Age>50 | 19 | .3721 | .5690 | .9205 | .8101 |
|  | 12 | .5046 | .7685 | .8728 | .7063 |
| Arrythmia | 19 | .4878 | .6166 | .8709 | .7138 |
|  | 12 | .6596 | .7927 | .7985 | .5691 |
| Stroke | 19 | .4896 | .7286 | .8687 | .7436 |
|  | 12 | .6624 | .8714 | .7953 | .5983 |
| Charlson>0 | 19 | .4572 | .6497 | .8808 | .7527 |
|  | 12 | .6372 | .7969 | .8113 | .6351 |
| CHF | 19 | .4850 | .5888 | .8739 | .7019 |
|  | 12 | .6557 | .7781 | .8038 | .5231 |
| COPD | 19 | .4821 | .5604 | .8760 | .8019 |
|  | 12 | .6519 | .7466 | .8072 | .6858 |
| Diabetes | 19 | .4208 | .6944 | .8938 | .7234 |
|  | 12 | .6139 | .8159 | .8226 | .6309 |
| Hypertension | 19 | .4176 | .5551 | .9045 | .8001 |
|  | 12 | .5802 | .7350 | .8450 | .6995 |
| Income quintile: high | 19 | .4879 | .5024 | .8665 | .8694 |
|  | 12 | .6616 | .6720 | .7888 | .7998 |
| Male | 19 | .5660 | .4681 | .8848 | .8530 |
|  | 12 | .7398 | .6397 | .8166 | .7739 |
| Rural location | 19 | .4846 | .5435 | .8703 | .8510 |
|  | 12 | .6618 | .6879 | .7965 | .7757 |

**Table S7**

**The RECORD statement – checklist of items, extended from the STROBE statement, that should be reported in observational studies using routinely collected health data.**

|  | **Item No.** | **STROBE items** | **Location in manuscript where items are reported** | **RECORD items** | **Location in manuscript where items are reported** |
| --- | --- | --- | --- | --- | --- |
| **Title and abstract** | | | | | |
|  | 1 | (a) Indicate the study’s design with a commonly used term in the title or the abstract (b) Provide in the abstract an informative and balanced summary of what was done and what was found | 1 | RECORD 1.1: The type of data used should be specified in the title or abstract. When possible, the name of the databases used should be included.  RECORD 1.2: If applicable, the geographic region and timeframe within which the study took place should be reported in the title or abstract.  RECORD 1.3: If linkage between databases was conducted for the study, this should be clearly stated in the title or abstract. | 1  2  2 |
| **Introduction** | | | | | |
| Background rationale | 2 | Explain the scientific background and rationale for the investigation being reported | 8 |  |  |
| Objectives | 3 | State specific objectives, including any prespecified hypotheses | 9/10 |  |  |
| **Methods** | | | | | |
| Study Design | 4 | Present key elements of study design early in the paper | 9/10 |  |  |
| Setting | 5 | Describe the setting, locations, and relevant dates, including periods of recruitment, exposure, follow-up, and data collection | 9/10 |  |  |
| Participants | 6 | *(a) Cohort study* - Give the eligibility criteria, and the sources and methods of selection of participants. Describe methods of follow-up  *Case-control study* - Give the eligibility criteria, and the sources and methods of case ascertainment and control selection. Give the rationale for the choice of cases and controls  *Cross-sectional study* - Give the eligibility criteria, and the sources and methods of selection of participants  *(b) Cohort study* - For matched studies, give matching criteria and number of exposed and unexposed  *Case-control study* - For matched studies, give matching criteria and the number of controls per case | 9/10 | RECORD 6.1: The methods of study population selection (such as codes or algorithms used to identify subjects) should be listed in detail. If this is not possible, an explanation should be provided.  RECORD 6.2: Any validation studies of the codes or algorithms used to select the population should be referenced. If validation was conducted for this study and not published elsewhere, detailed methods and results should be provided.  RECORD 6.3: If the study involved linkage of databases, consider use of a flow diagram or other graphical display to demonstrate the data linkage process, including the number of individuals with linked data at each stage. | 11  11 |
| Variables | 7 | Clearly define all outcomes, exposures, predictors, potential confounders, and effect modifiers. Give diagnostic criteria, if applicable. | 10/11 | RECORD 7.1: A complete list of codes and algorithms used to classify exposures, outcomes, confounders, and effect modifiers should be provided. If these cannot be reported, an explanation should be provided. | 11 |
| Data sources/ measurement | 8 | For each variable of interest, give sources of data and details of methods of assessment (measurement).  Describe comparability of assessment methods if there is more than one group | 10/11 |  |  |
| Bias | 9 | Describe any efforts to address potential sources of bias | 11 |  |  |
| Study size | 10 | Explain how the study size was arrived at | 11 |  |  |
| Quantitative variables | 11 | Explain how quantitative variables were handled in the analyses. If applicable, describe which groupings were chosen, and why | 11 |  |  |
| Statistical methods | 12 | (a) Describe all statistical methods, including those used to control for confounding  (b) Describe any methods used to examine subgroups and interactions  (c) Explain how missing data were addressed  (d) *Cohort study* - If applicable, explain how loss to follow-up was addressed  *Case-control study* - If applicable, explain how matching of cases and controls was addressed  *Cross-sectional study* - If applicable, describe analytical methods taking account of sampling strategy  (e) Describe any sensitivity analyses | 11-13 |  |  |
| Data access and cleaning methods |  | .. |  | RECORD 12.1: Authors should describe the extent to which the investigators had access to the database population used to create the study population.  RECORD 12.2: Authors should provide information on the data cleaning methods used in the study. | 10-11  12 |
| Linkage |  | .. |  | RECORD 12.3: State whether the study included person-level, institutional-level, or other data linkage across two or more databases. The methods of linkage and methods of linkage quality evaluation should be provided. | 10 |
| **Results** | | | | | |
| Participants | 13 | (a) Report the numbers of individuals at each stage of the study (*e.g.*, numbers potentially eligible, examined for eligibility, confirmed eligible, included in the study, completing follow-up, and analysed)  (b) Give reasons for non-participation at each stage.  (c) Consider use of a flow diagram | 12 | RECORD 13.1: Describe in detail the selection of the persons included in the study (*i.e.,* study population selection) including filtering based on data quality, data availability and linkage. The selection of included persons can be described in the text and/or by means of the study flow diagram. | 10-12 |
| Descriptive data | 14 | (a) Give characteristics of study participants (*e.g.*, demographic, clinical, social) and information on exposures and potential confounders  (b) Indicate the number of participants with missing data for each variable of interest  (c) *Cohort study* - summarise follow-up time (*e.g.*, average and total amount) | 12-13 |  |  |
| Outcome data | 15 | *Cohort study* - Report numbers of outcome events or summary measures over time  *Case-control study* - Report numbers in each exposure category, or summary measures of exposure  *Cross-sectional study* - Report numbers of outcome events or summary measures | 15 |  |  |
| Main results | 16 | (a) Give unadjusted estimates and, if applicable, confounder-adjusted estimates and their precision (e.g., 95% confidence interval). Make clear which confounders were adjusted for and why they were included  (b) Report category boundaries when continuous variables were categorized  (c) If relevant, consider translating estimates of relative risk into absolute risk for a meaningful time period | 15/16 |  |  |
| Other analyses | 17 | Report other analyses done—e.g., analyses of subgroups and interactions, and sensitivity analyses | 16 |  |  |
| **Discussion** | | | | | |
| Key results | 18 | Summarise key results with reference to study objectives | 17/18 |  |  |
| Limitations | 19 | Discuss limitations of the study, taking into account sources of potential bias or imprecision. Discuss both direction and magnitude of any potential bias | 17/18 | RECORD 19.1: Discuss the implications of using data that were not created or collected to answer the specific research question(s). Include discussion of misclassification bias, unmeasured confounding, missing data, and changing eligibility over time, as they pertain to the study being reported. | 19-20 |
| Interpretation | 20 | Give a cautious overall interpretation of results considering objectives, limitations, multiplicity of analyses, results from similar studies, and other relevant evidence | 19 |  |  |
| Generalisability | 21 | Discuss the generalisability (external validity) of the study results | 19/20 |  |  |
| **Other Information** | | | | | |
| Funding | 22 | Give the source of funding and the role of the funders for the present study and, if applicable, for the original study on which the present article is based | 3 |  |  |
| Accessibility of protocol, raw data, and programming code |  | .. |  | RECORD 22.1: Authors should provide information on how to access any supplemental information such as the study protocol, raw data, or programming code. | 3-4 |

*Reference: Benchimol EI, Smeeth L, Guttmann A, Harron K, Moher D, Petersen I, Sørensen HT, von Elm E, Langan SM, the RECORD Working Committee. The REporting of studies Conducted using Observational Routinely-collected health Data (RECORD) Statement. *PLoS Medicine* 2015; in press.

*Checklist is protected under Creative Commons Attribution ([CC BY](http://creativecommons.org/licenses/by/4.0/)) license.

**Table S8: Bootstrap Imputation Approach (BIA) Schematic and Previous Study Overview Table**

| Peer-reviewed Study DOI | Disease or Procedure | Probability Model Performance | Misclassification Adjustment |
| --- | --- | --- | --- |
| https://doi.org/10.1016/j.jclinepi.2017.12.012 | Colles Fracture | c-statistic= 98.9 calibration slope = 1.009 | - Relative bias in disease prevalence BIA vs gold-standard=0.01% - Across 8 covariates (age, sex, diagnostic risk etc) misclassification bias for measures of association was significantly larger with the ICD code vs. BI |
| <https://doi.org/10.1097/mlr.0000000000000787>  https://doi.org/10.1016/j.jclinepi.2017.01.007 | Severe Renal Failure (defined as a glomerular filtration rate less than 30 mL/min/1.73m^2^ at any time during the hospitalization) | c-statistic 0.937  Hosmer–Lemeshow statistic= 48.5 | - In this large cohort of hospitalized patients, QBA increased misclassification bias. BI decreased misclassification bias even without collecting additional information |
| <https://doi.org/10.1016/j.jclinepi.2017.01.007>  10.1097/MD.0000000000038238 | Acetabular Fracture | Optimism-corrected: c-statistic 0.988, calibration slope 1.06  External validation:  c-statistic 0.90, 95% CI: 0.87-0.92) and very well calibrated (integrated calibration index 0.056, 95% CI: 0.039-0.074 | When model-based expected probabilities were used to determine AF status using bootstrap imputation methods, misclassification bias for AF prevalence and its association with other variables was much lower than with International Classification of Disease, 10^th^ revision S32.4 (median [range] relative difference 1.0% [0%–9.0%] vs 18.0% [5.4%–75.0% |
| 10.1186/s12874-024-02199-1 | Cystectomy | c-statistic [C] 0.999, Integrated Calibration Index [ICI] 0.000) | Misclassification bias was minimized when model-based based predictions were used to probabilistically impute cystectomy-diversion status across bootstrap resamples |

S-References:

1.	Kendzerska T, van Walraven C, McIsaac DI, Povitz M, Mulpuru S, Lima I, Talarico R, Aaron SD, Reisman W, Gershon AS: **Case-Ascertainment Models to Identify Adults with Obstructive Sleep Apnea Using Health Administrative Data: Internal and External Validation**. *Clinical epidemiology* 2021, **13**:453-467.
