# Supplementary material for: A population-based study of obstructive sleep apnea: using probabilistic case ascertainment through health administrative databases
Source: Int J Epidemiol. 2026 Aug 20;55(5):dyag176. doi: 10.1093/ije/dyag176 (PMC13493051; doi:10.1093/ije/dyag176)
Supplement: Supplementary file 1 [file dyag176_supplementary_data.zip › ije-2026-01-0177-File007.pdf]

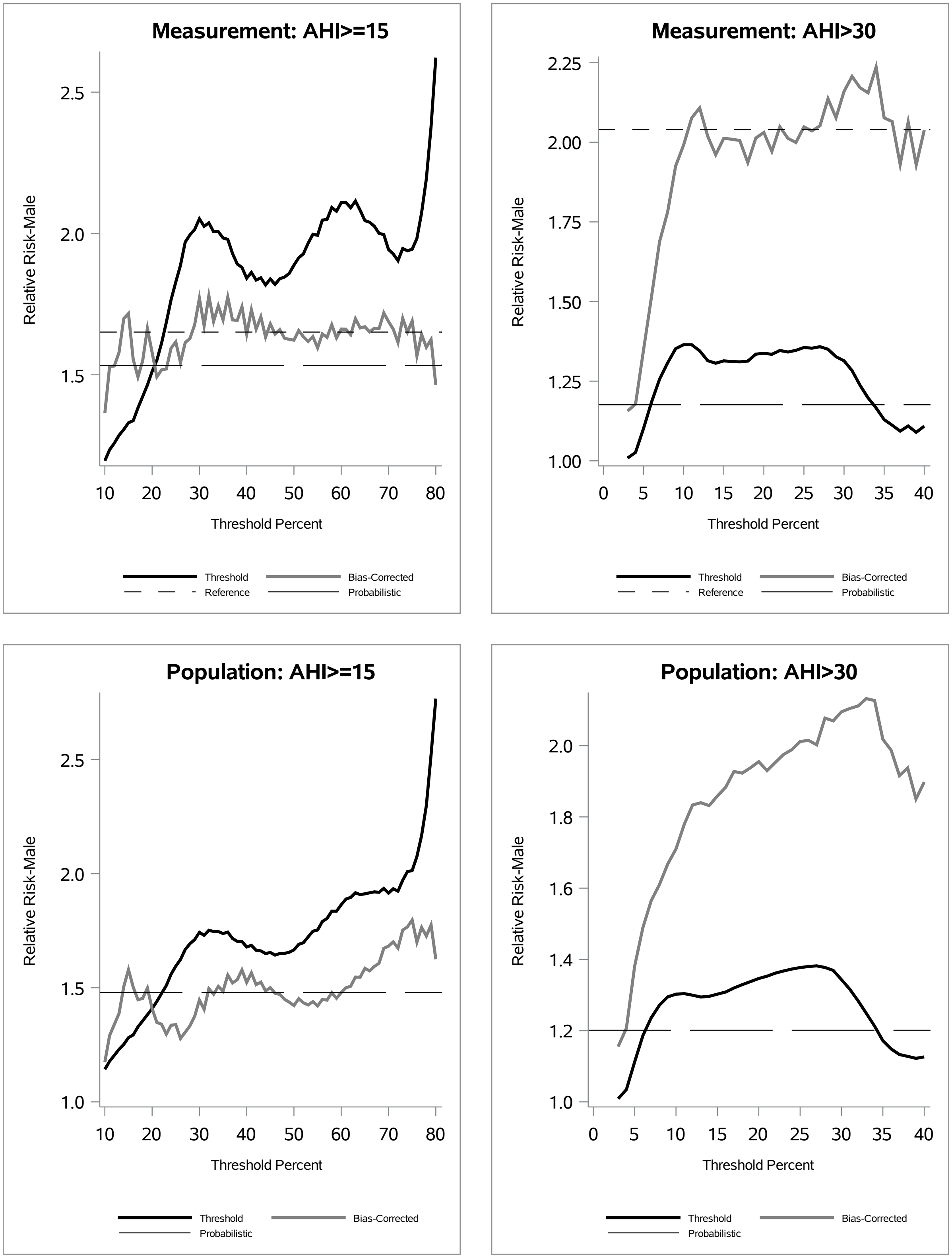
Measurement: AHI>=15
Relative Risk-Male
Threshold Percent
2.5
2.0
1.5
10 20 30 40 50 60 70 80
Threshold
Bias-Corrected
Reference
Probabilistic
Measurement: AHI>30
2.25
2.00
1.75
1.50
1.25
1.00
0 5 10 15 20 25 30 35 40
Population: AHI>=15
1.0
Population: AHI>30
2.0
1.8
1.6
1.4
1.2
1.0

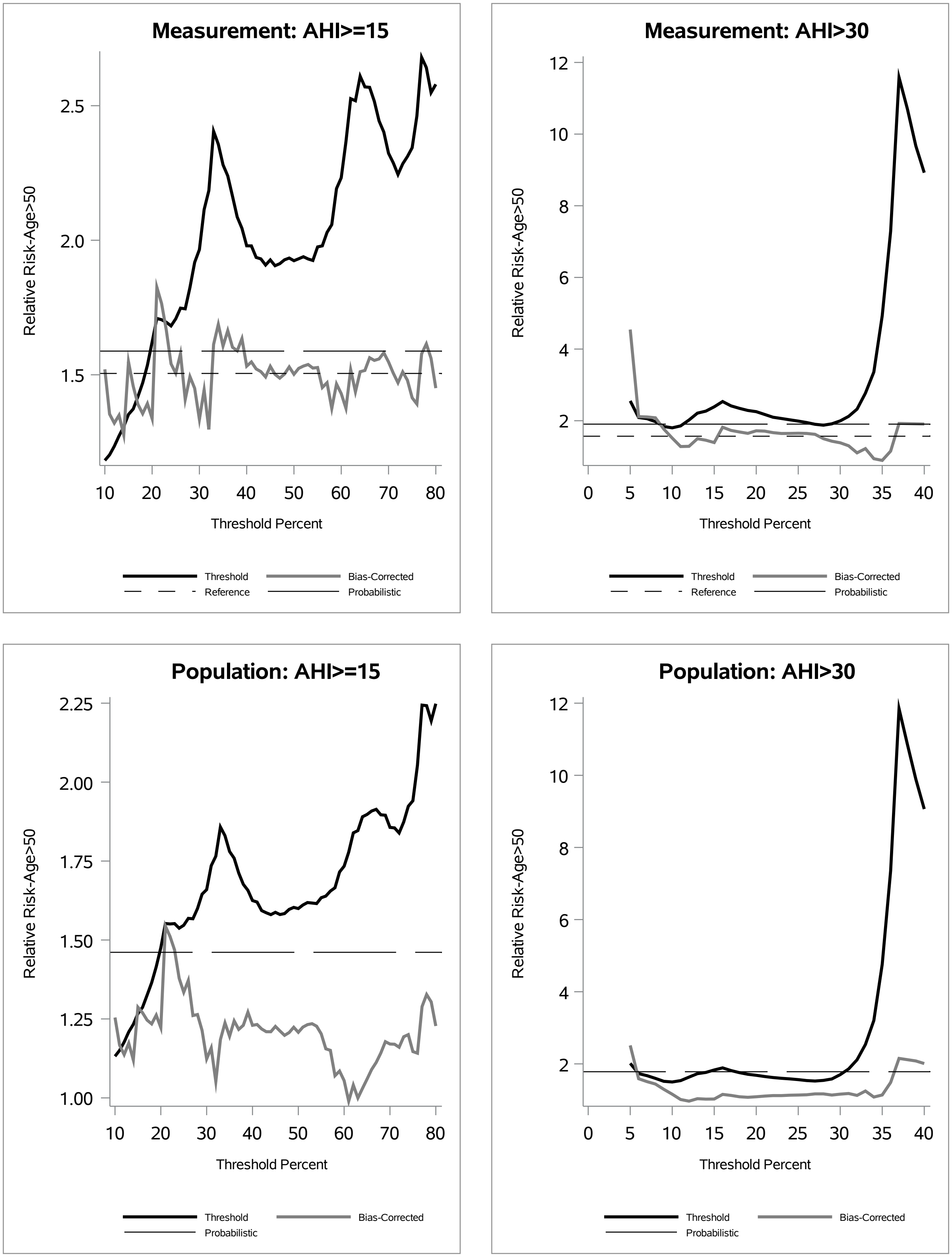

Measurement: AHI>=15
Relative Risk-Age>50
Threshold Percent
2.5
2.0
1.5
10 20 30 40 50 60 70 80
Threshold
Bias-Corrected
Reference
Probabilistic
Measurement: AHI>30
Relative Risk-Age>50
Threshold Percent
12
10
8
6
4
2
0 5 10 15 20 25 30 35 40
Threshold
Bias-Corrected
Reference
Probabilistic
Population: AHI>=15
Relative Risk-Age>50
Threshold Percent
2.25
2.00
1.75
1.50
1.25
1.00
10 20 30 40 50 60 70 80
Threshold
Bias-Corrected
Probabilistic
Population: AHI>30
Relative Risk-Age>50
Threshold Percent
12
10
8
6
4
2
0 5 10 15 20 25 30 35 40
Threshold
Bias-Corrected
Probabilistic

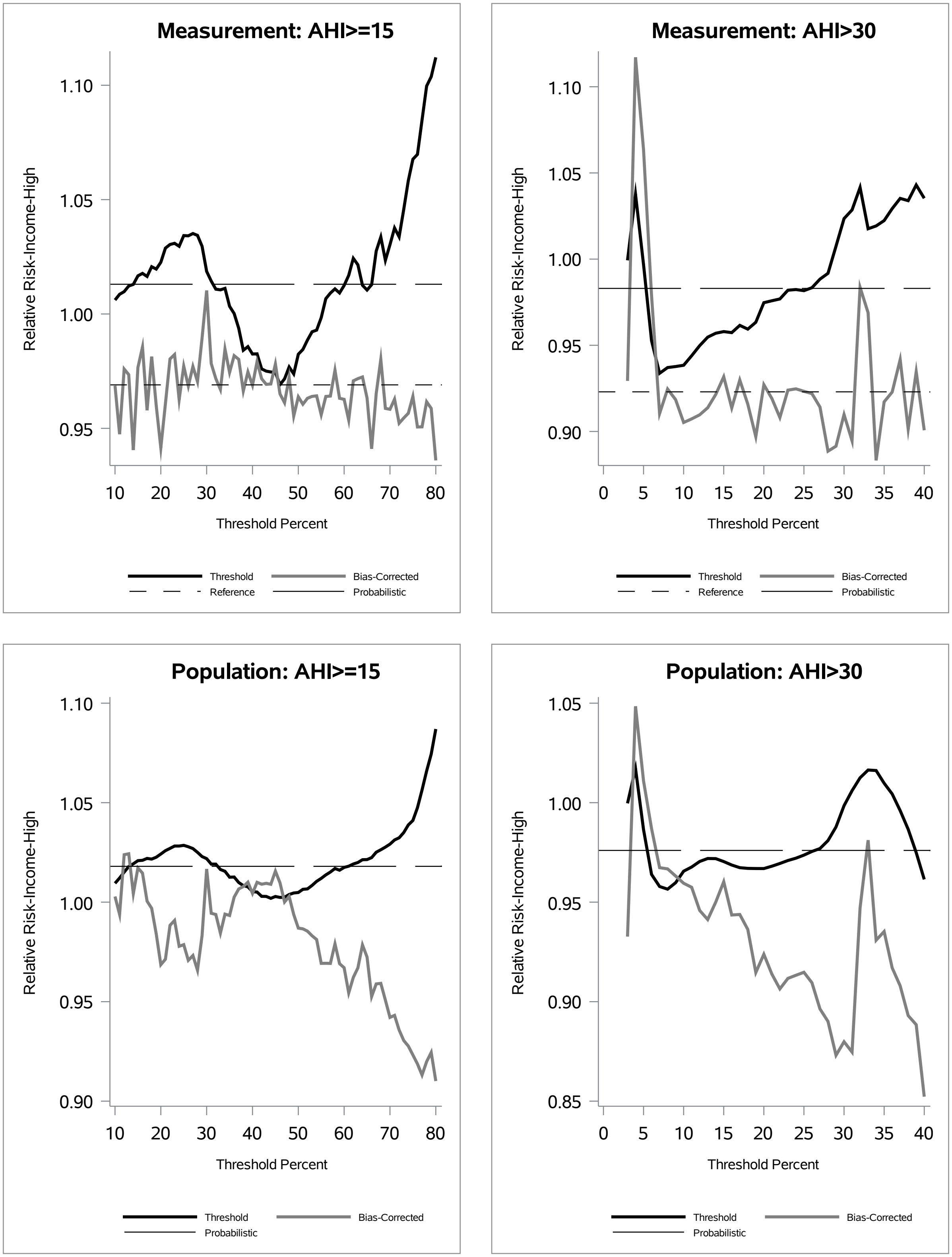
Measurement: AHI>=15
Relative Risk-Income-High
1.10
1.05
1.00
0.95
10 20 30 40 50 60 70 80
Threshold Percent
Threshold
Bias-Corrected
Reference
Probabilistic
Measurement: AHI>30
Relative Risk-Income-High
1.10
1.05
1.00
0.95
0.90
0 5 10 15 20 25 30 35 40
Threshold Percent
Threshold
Bias-Corrected
Reference
Probabilistic
Population: AHI>=15
Relative Risk-Income-High
1.10
1.05
1.00
0.95
0.90
10 20 30 40 50 60 70 80
Threshold Percent
Threshold
Bias-Corrected
Probabilistic
Population: AHI>30
Relative Risk-Income-High
1.05
1.00
0.95
0.90
0.85
0 5 10 15 20 25 30 35 40
Threshold Percent
Threshold
Bias-Corrected
Probabilistic

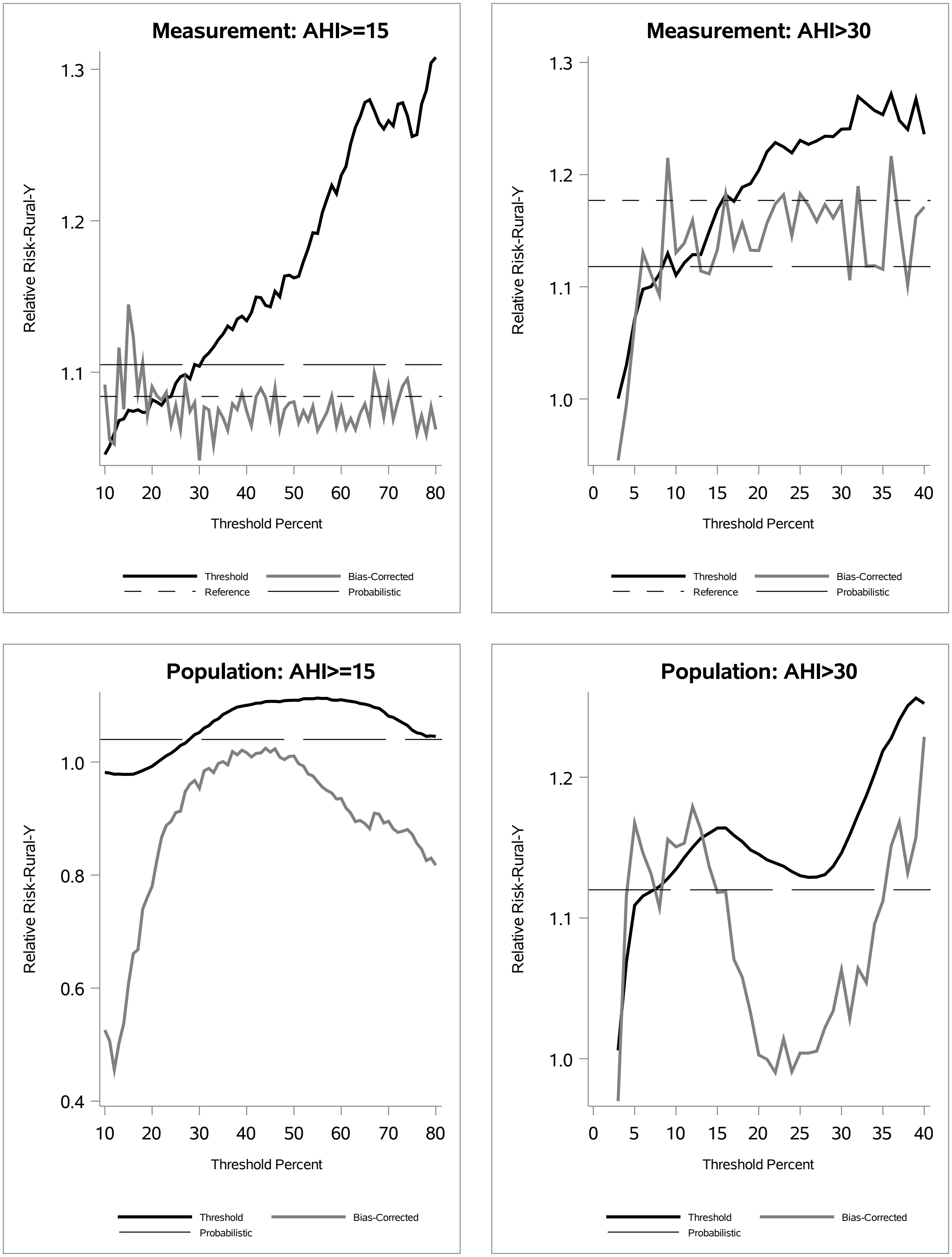
Measurement: AHI>=15
Relative Risk-Rural-Y
Threshold Percent
Threshold
Bias-Corrected
Reference
Probabilistic
Measurement: AHI>30
Relative Risk-Rural-Y
Threshold Percent
Threshold
Bias-Corrected
Reference
Probabilistic
Population: AHI>=15
Relative Risk-Rural-Y
Threshold Percent
Threshold
Bias-Corrected
Probabilistic
Population: AHI>30
Relative Risk-Rural-Y
Threshold Percent
Threshold
Bias-Corrected
Probabilistic

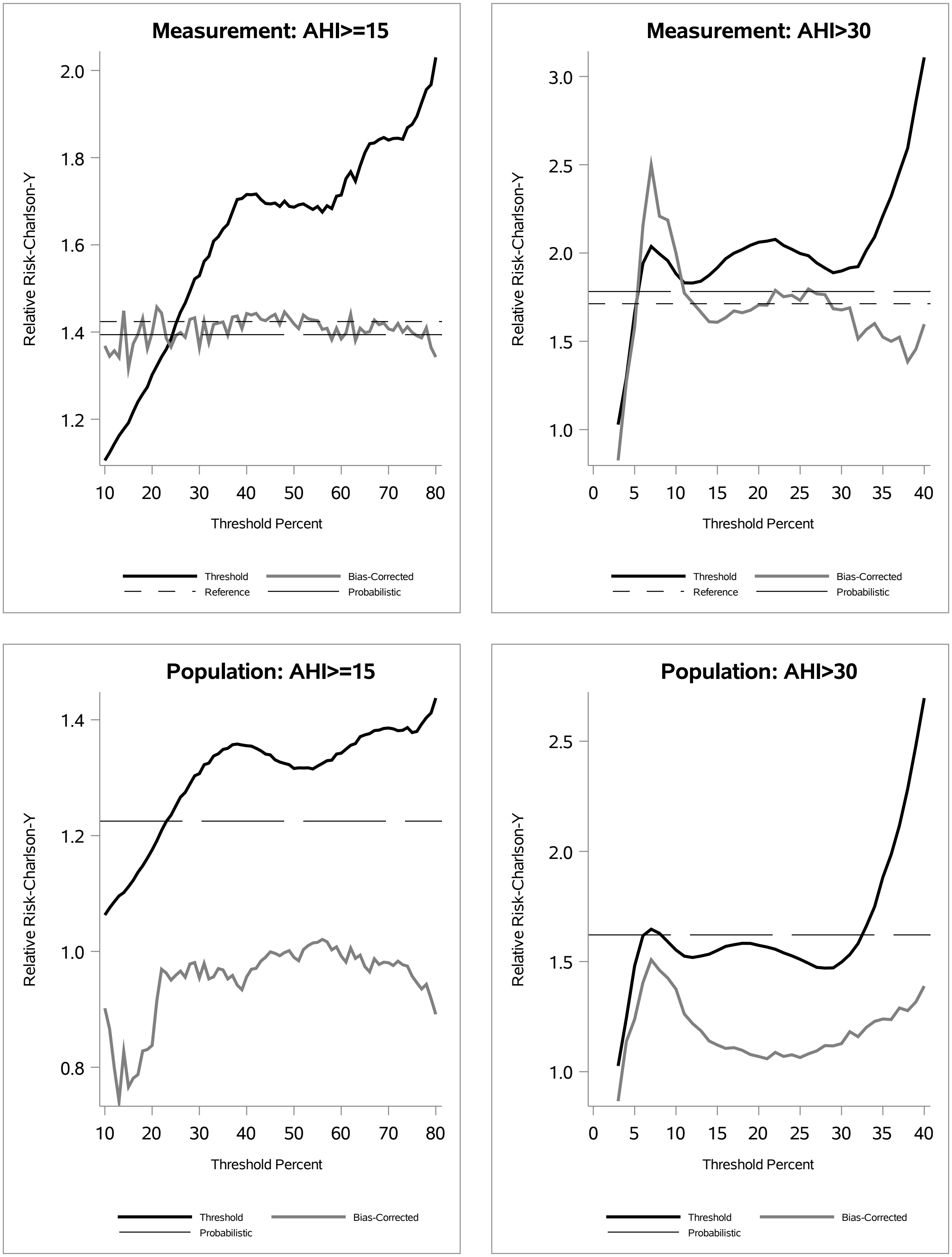

Measurement: AHI>=15
Relative Risk-Charlson-Y
2.0
1.8
1.6
1.4
1.2
10 20 30 40 50 60 70 80
Threshold Percent
Threshold
Bias-Corrected
Reference
Probabilistic
Measurement: AHI>30
Relative Risk-Charlson-Y
3.0
2.5
2.0
1.5
1.0
0 5 10 15 20 25 30 35 40
Threshold Percent
Threshold
Bias-Corrected
Reference
Probabilistic
Population: AHI>=15
Relative Risk-Charlson-Y
1.4
1.2
1.0
0.8
10 20 30 40 50 60 70 80
Threshold Percent
Threshold
Bias-Corrected
Probabilistic
Population: AHI>30
Relative Risk-Charlson-Y
2.5
2.0
1.5
1.0
0 5 10 15 20 25 30 35 40
Threshold Percent
Threshold
Bias-Corrected
Probabilistic

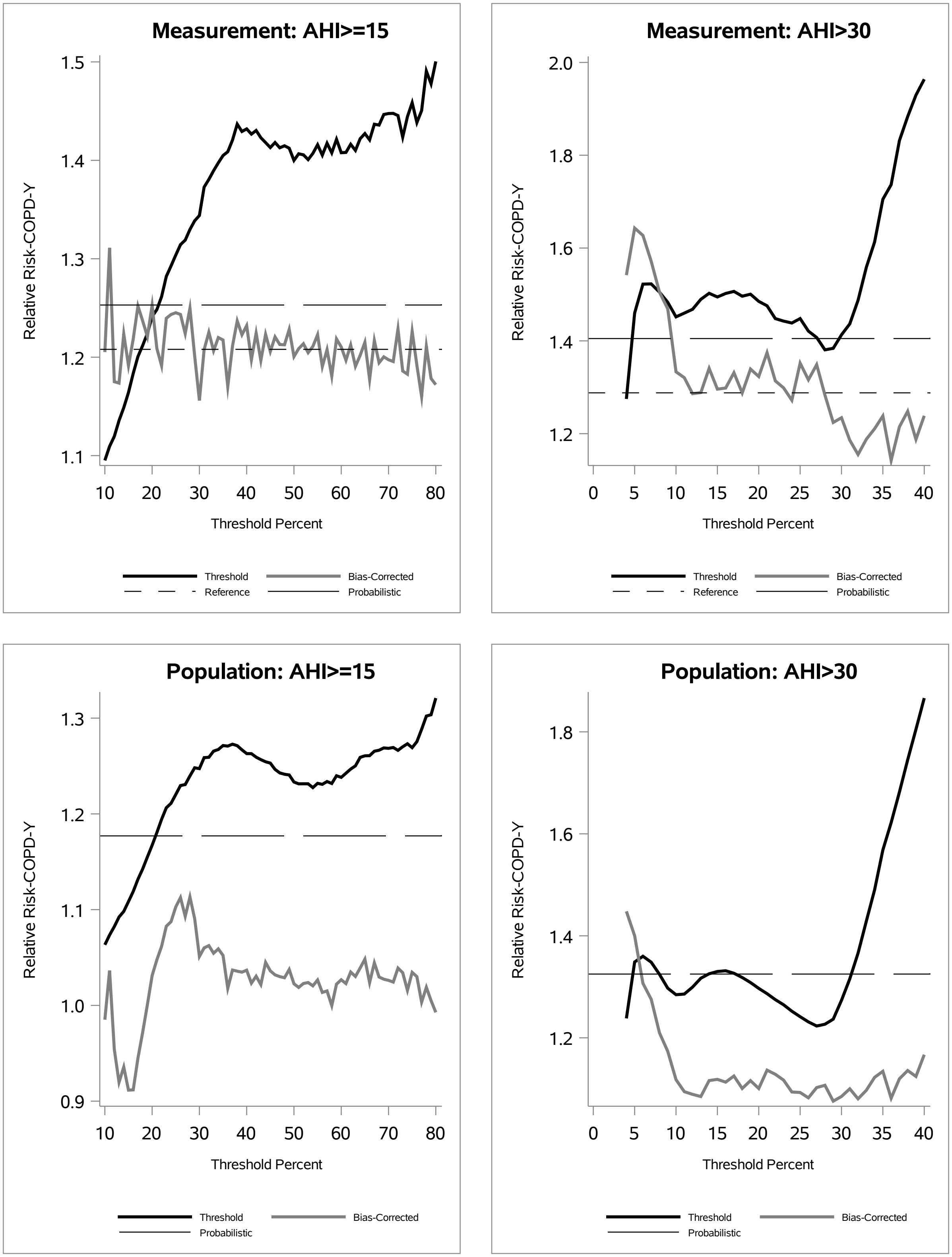
Measurement: AHI>=15
Relative Risk-COPD-Y
Threshold Percent
Threshold
Bias-Corrected
Reference
Probabilistic
Measurement: AHI>30
Relative Risk-COPD-Y
Threshold Percent
Threshold
Bias-Corrected
Reference
Probabilistic
Population: AHI>=15
Relative Risk-COPD-Y
Threshold Percent
Threshold
Bias-Corrected
Probabilistic
Population: AHI>30
Relative Risk-COPD-Y
Threshold Percent
Threshold
Bias-Corrected
Probabilistic

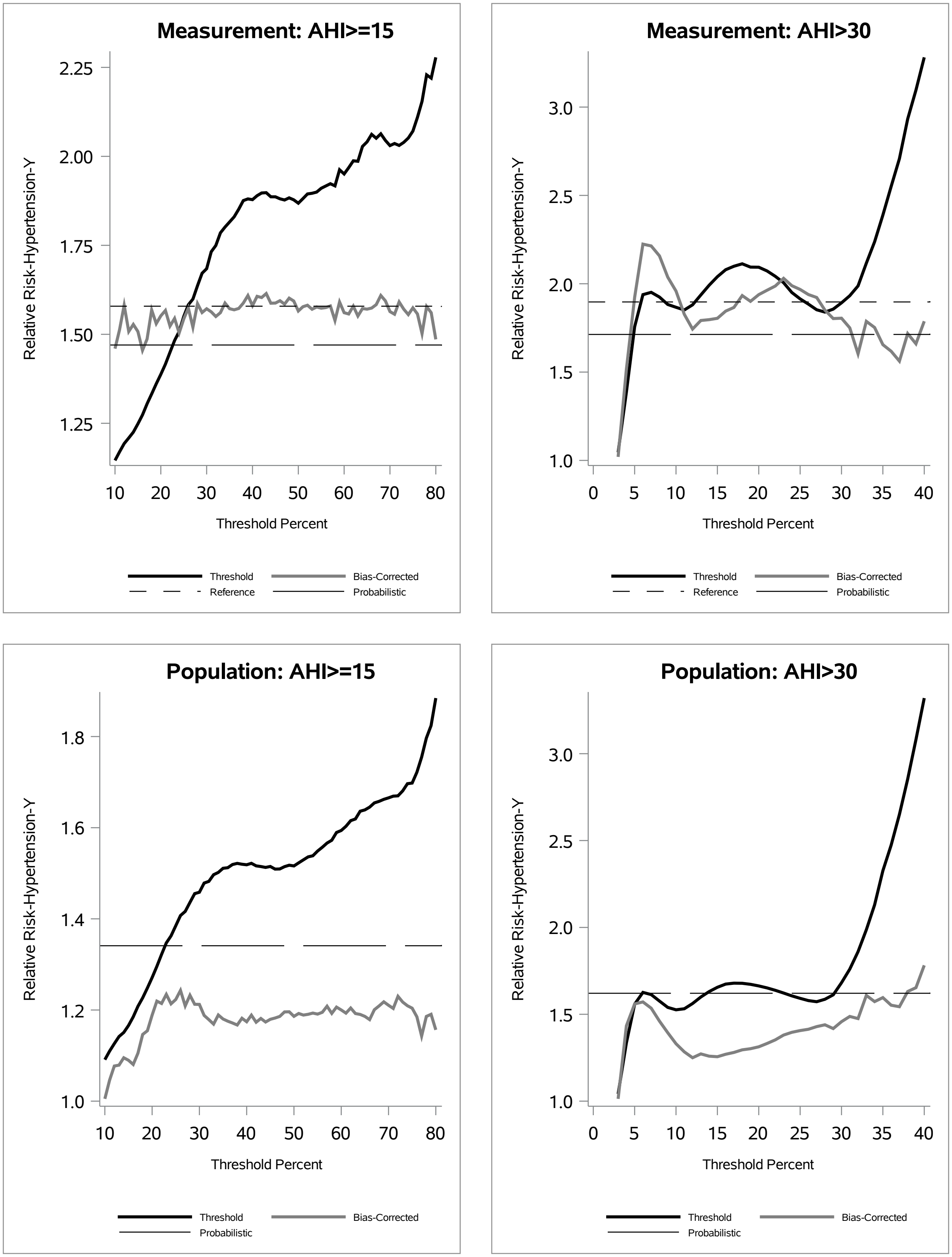

Measurement: AHI>=15
Measurement: AHI>30
Population: AHI>=15
Population: AHI>30
Relative Risk-Hypertension-Y
Threshold Percent
Threshold
Bias-Corrected
Reference
Probabilistic

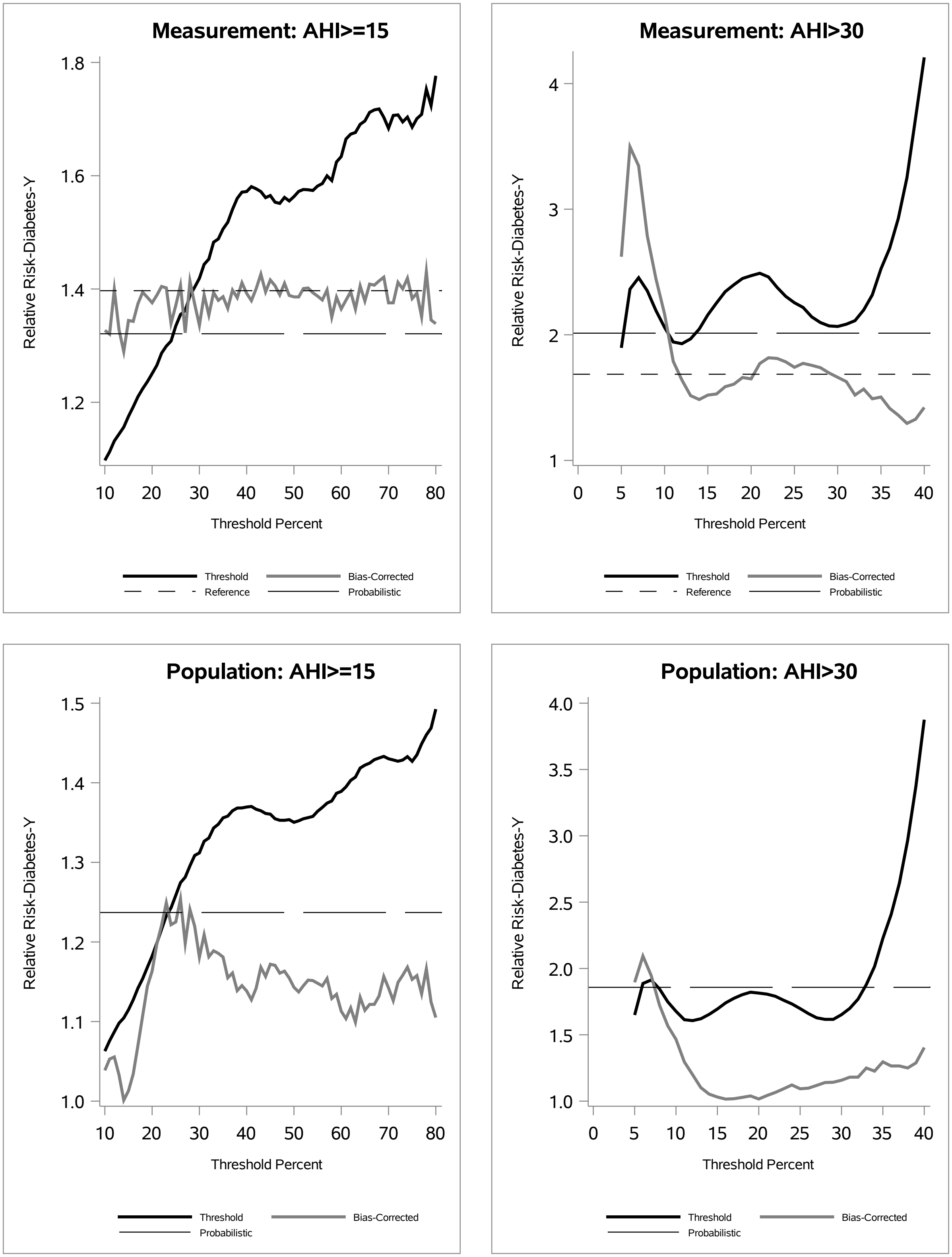
Measurement: AHI>=15
Relative Risk-Diabetes-Y
1.8
1.6
1.4
1.2
10 20 30 40 50 60 70 80
Threshold Percent
Threshold
Bias-Corrected
Reference
Probabilistic
Measurement: AHI>30
Relative Risk-Diabetes-Y
4
3
2
1
0 5 10 15 20 25 30 35 40
Threshold Percent
Threshold
Bias-Corrected
Reference
Probabilistic
Population: AHI>=15
Relative Risk-Diabetes-Y
1.5
1.4
1.3
1.2
1.1
1.0
10 20 30 40 50 60 70 80
Threshold Percent
Threshold
Bias-Corrected
Probabilistic
Population: AHI>30
Relative Risk-Diabetes-Y
4.0
3.5
3.0
2.5
2.0
1.5
1.0
0 5 10 15 20 25 30 35 40
Threshold Percent
Threshold
Bias-Corrected
Probabilistic

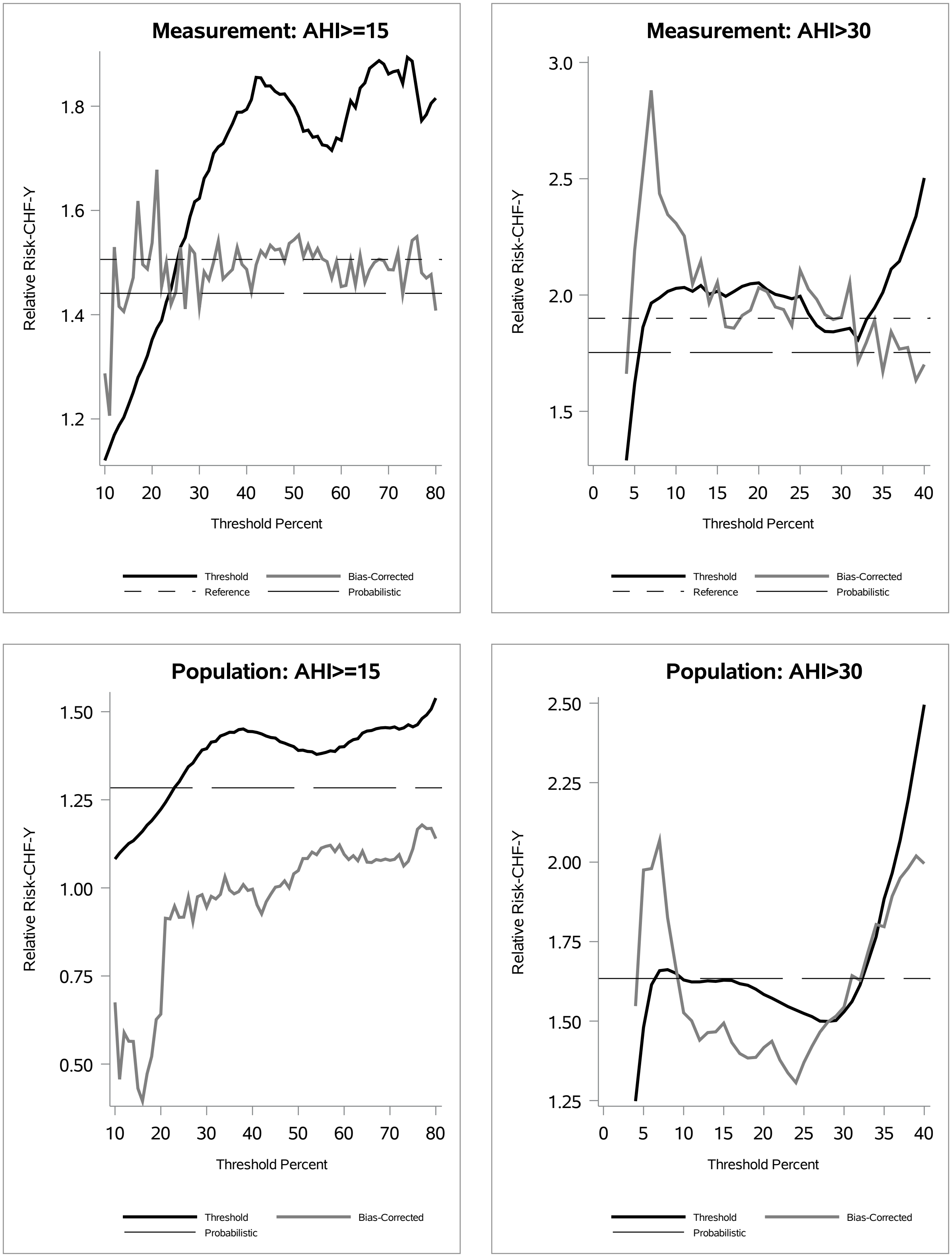
Measurement: AHI>=15
Relative Risk-CHF-Y
1.2
1.4
1.6
1.8
10 20 30 40 50 60 70 80
Threshold Percent
Threshold
Bias-Corrected
Reference
Probabilistic
Measurement: AHI>30
Relative Risk-CHF-Y
1.5
2.0
2.5
3.0
0 5 10 15 20 25 30 35 40
Threshold Percent
Threshold
Bias-Corrected
Reference
Probabilistic
Population: AHI>=15
Relative Risk-CHF-Y
0.50
0.75
1.00
1.25
1.50
10 20 30 40 50 60 70 80
Threshold Percent
Threshold
Bias-Corrected
Probabilistic
Population: AHI>30
Relative Risk-CHF-Y
1.25
1.50
1.75
2.00
2.25
2.50
0 5 10 15 20 25 30 35 40
Threshold Percent
Threshold
Bias-Corrected
Probabilistic

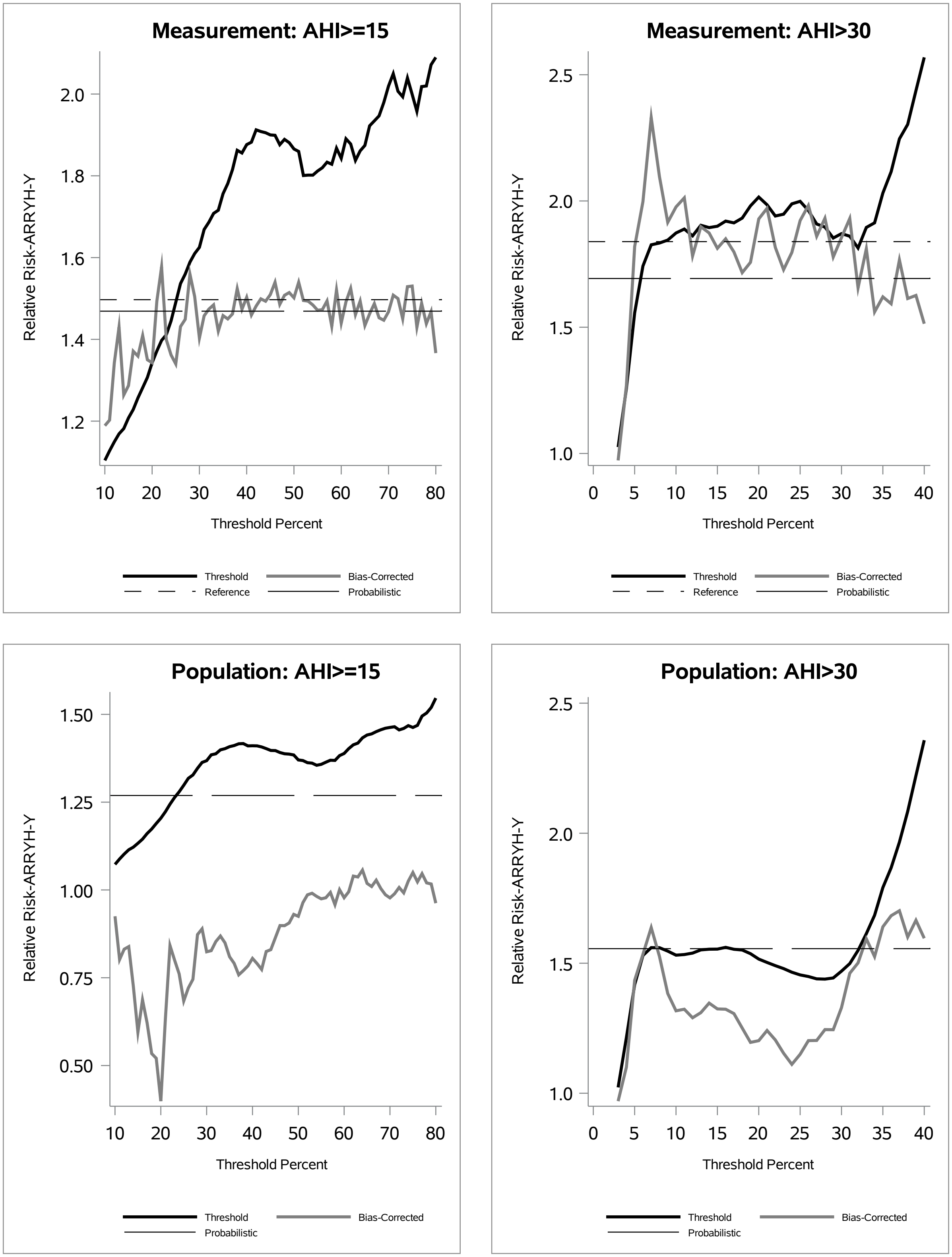
Measurement: AHI>=15
Relative Risk-ARRYH-Y
1.2
1.4
1.6
1.8
2.0
10 20 30 40 50 60 70 80
Threshold Percent
Threshold
Bias-Corrected
Reference
Probabilistic
Measurement: AHI>30
Relative Risk-ARRYH-Y
1.0
1.5
2.0
2.5
0 5 10 15 20 25 30 35 40
Threshold Percent
Threshold
Bias-Corrected
Reference
Probabilistic
Population: AHI>=15
Relative Risk-ARRYH-Y
0.50
0.75
1.00
1.25
1.50
10 20 30 40 50 60 70 80
Threshold Percent
Threshold
Bias-Corrected
Probabilistic
Population: AHI>30
Relative Risk-ARRYH-Y
1.0
1.5
2.0
2.5
0 5 10 15 20 25 30 35 40
Threshold Percent
Threshold
Bias-Corrected
Probabilistic

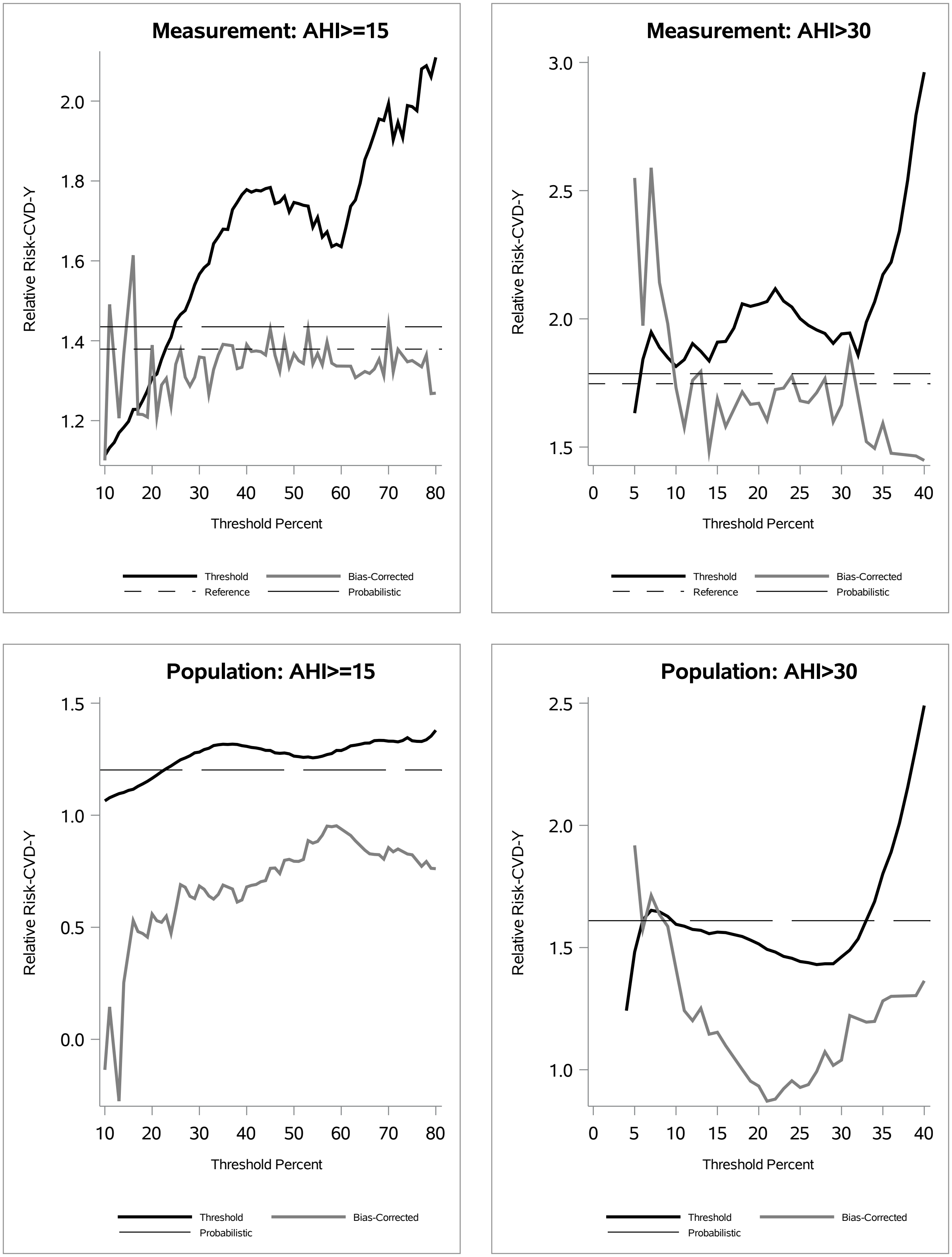
Measurement: AHI>=15
Measurement: AHI>30
Population: AHI>=15
Population: AHI>30
Relative Risk-CVD-Y
Threshold Percent
Threshold
Bias-Corrected
Reference
Probabilistic
