# Supplementary material for: A population-based study of obstructive sleep apnea: using probabilistic case ascertainment through health administrative databases
Source: Int J Epidemiol. 2026 Aug 20;55(5):dyag176. doi: 10.1093/ije/dyag176 (PMC13493051; doi:10.1093/ije/dyag176)
Supplement: Supplementary file 1 [file dyag176_supplementary_data.zip › ije-2026-01-0177-File008.pdf]

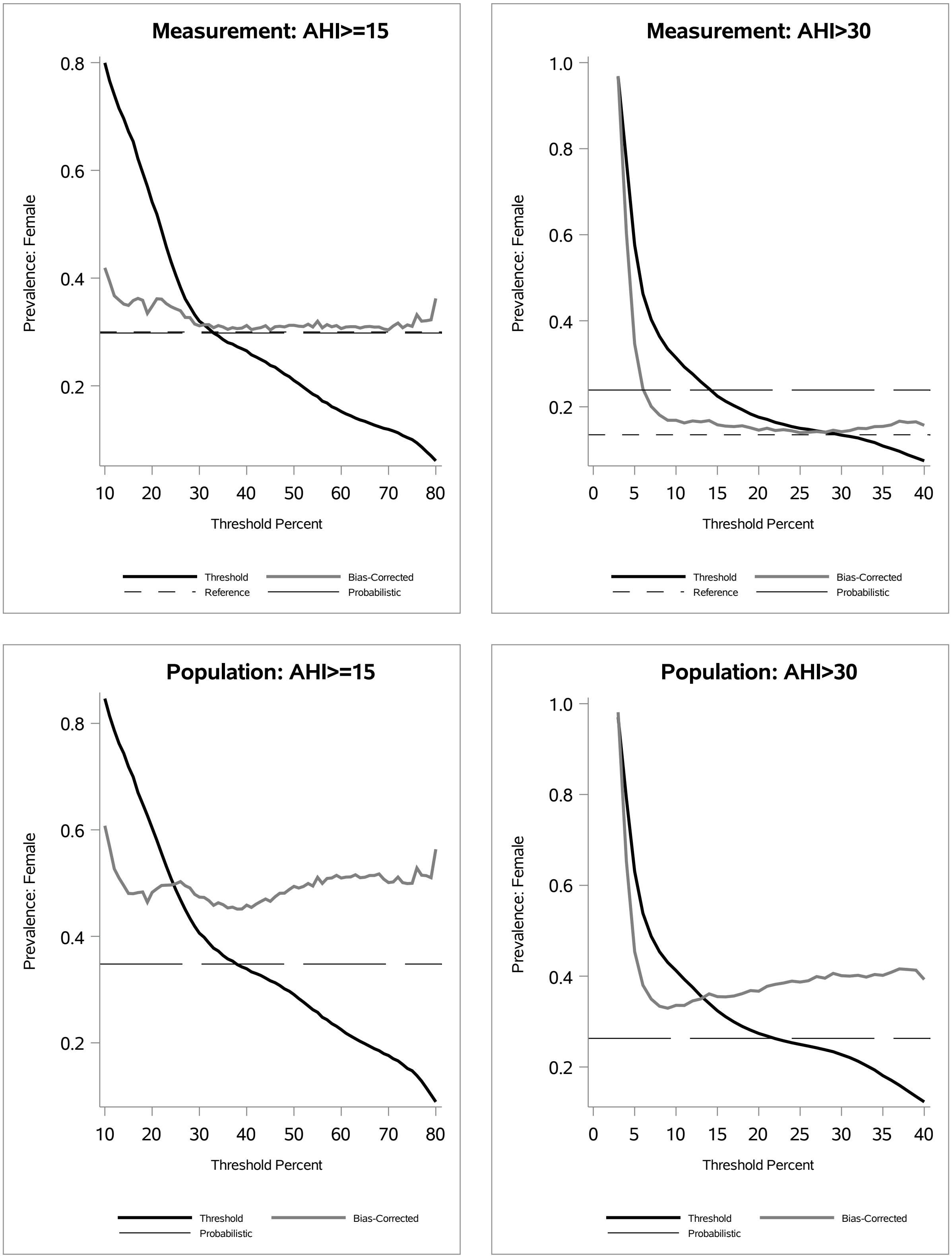
Measurement: AHI>=15
Prevalence: Female
Threshold Percent
Threshold
Bias-Corrected
Reference
Probabilistic
Measurement: AHI>30
Prevalence: Female
Threshold Percent
Threshold
Bias-Corrected
Reference
Probabilistic
Population: AHI>=15
Prevalence: Female
Threshold Percent
Threshold
Bias-Corrected
Probabilistic
Population: AHI>30
Prevalence: Female
Threshold Percent
Threshold
Bias-Corrected
Probabilistic

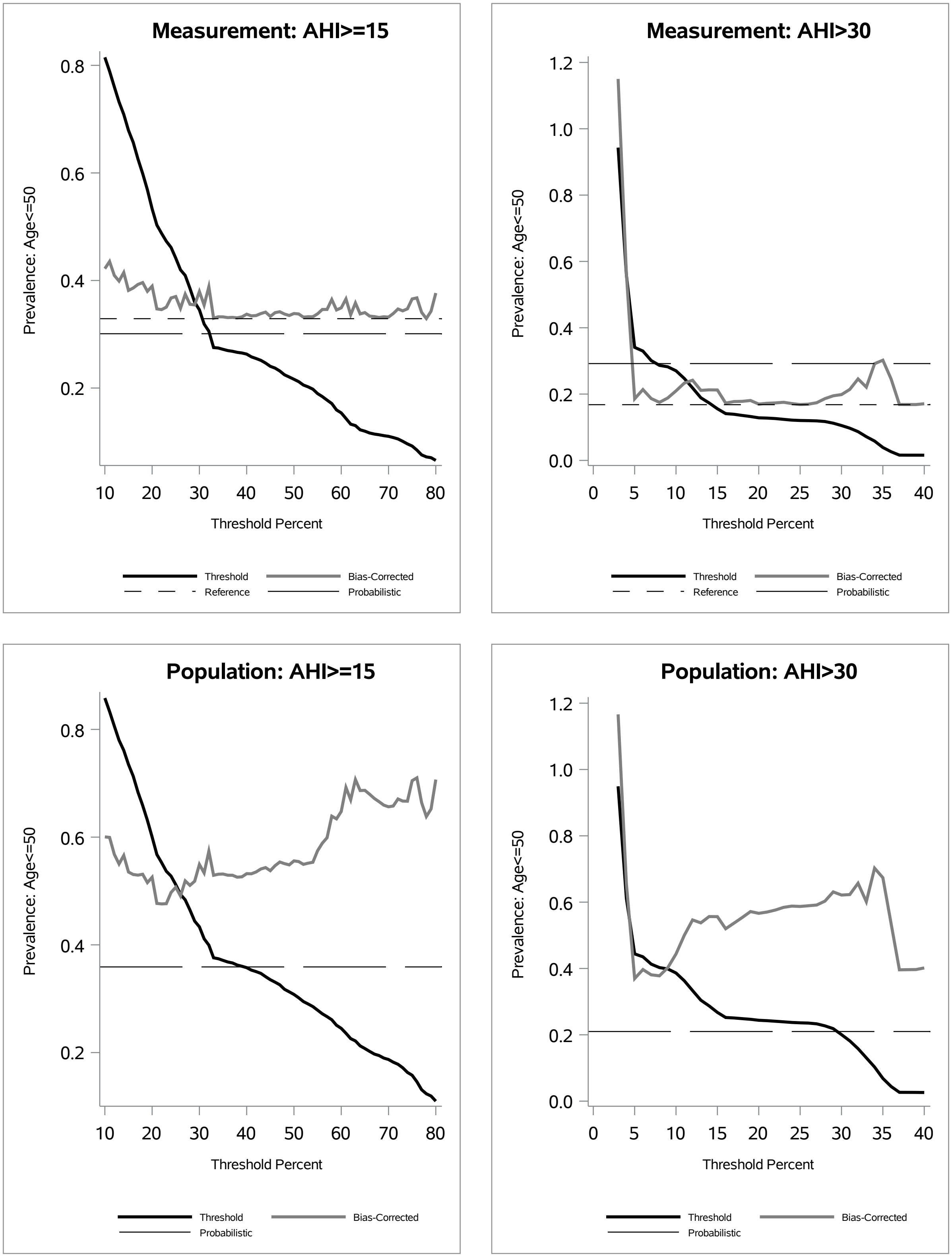
Measurement: AHI>=15
Prevalence: Age<=50
0.8
0.6
0.4
0.2
10 20 30 40 50 60 70 80
Threshold Percent
Threshold
Bias-Corrected
Reference
Probabilistic
Measurement: AHI>30
Prevalence: Age<=50
1.2
1.0
0.8
0.6
0.4
0.2
0.0
0 5 10 15 20 25 30 35 40
Threshold Percent
Threshold
Bias-Corrected
Reference
Probabilistic
Population: AHI>=15
Prevalence: Age<=50
0.8
0.6
0.4
0.2
10 20 30 40 50 60 70 80
Threshold Percent
Threshold
Bias-Corrected
Probabilistic
Population: AHI>30
Prevalence: Age<=50
1.2
1.0
0.8
0.6
0.4
0.2
0.0
0 5 10 15 20 25 30 35 40
Threshold Percent
Threshold
Bias-Corrected
Probabilistic

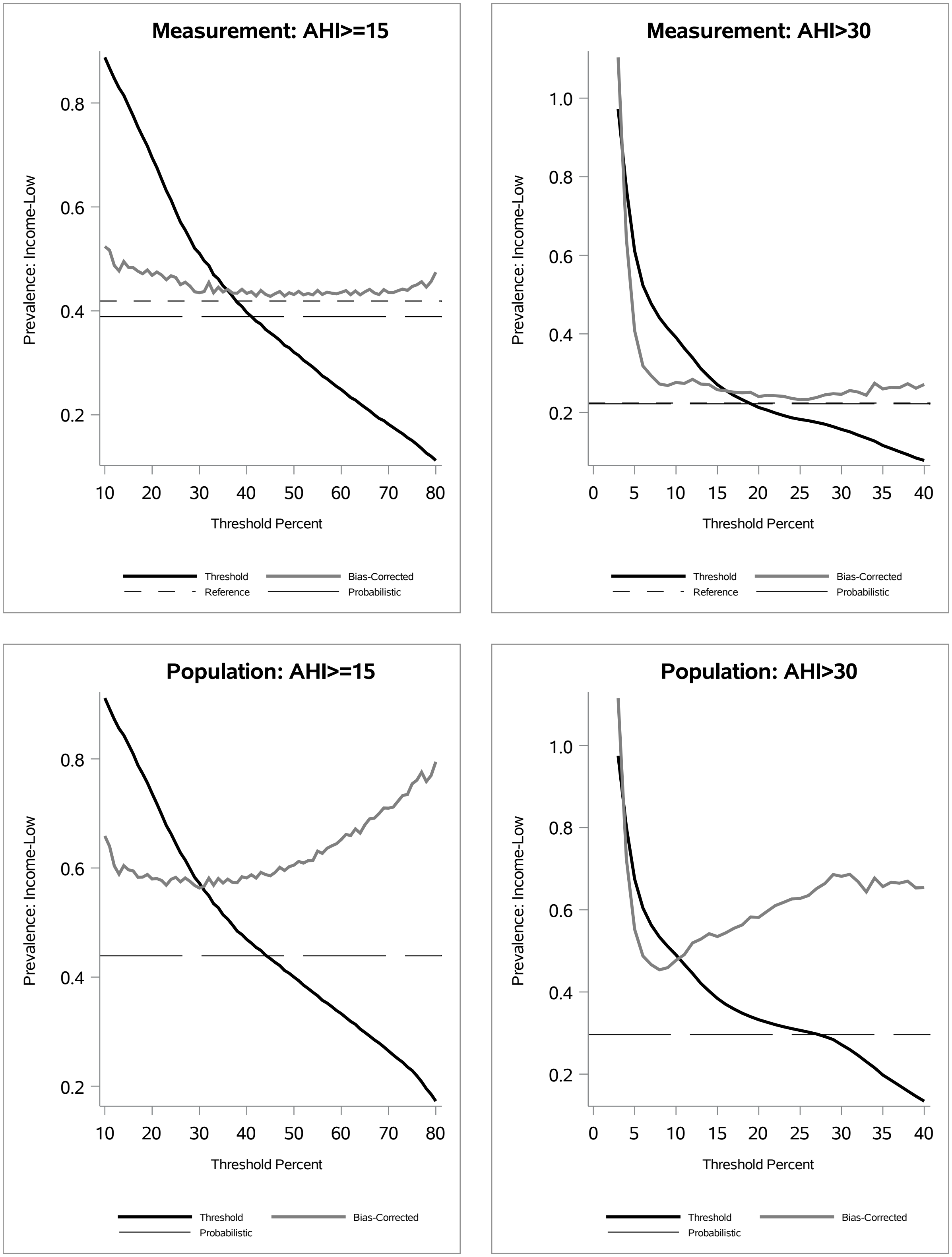
Measurement: AHI>=15
Prevalence: Income-Low
Threshold Percent
Threshold
Bias-Corrected
Reference
Probabilistic
Measurement: AHI>30
Prevalence: Income-Low
Threshold Percent
Threshold
Bias-Corrected
Reference
Probabilistic
Population: AHI>=15
Prevalence: Income-Low
Threshold Percent
Threshold
Bias-Corrected
Probabilistic
Population: AHI>30
Prevalence: Income-Low
Threshold Percent
Threshold
Bias-Corrected
Probabilistic

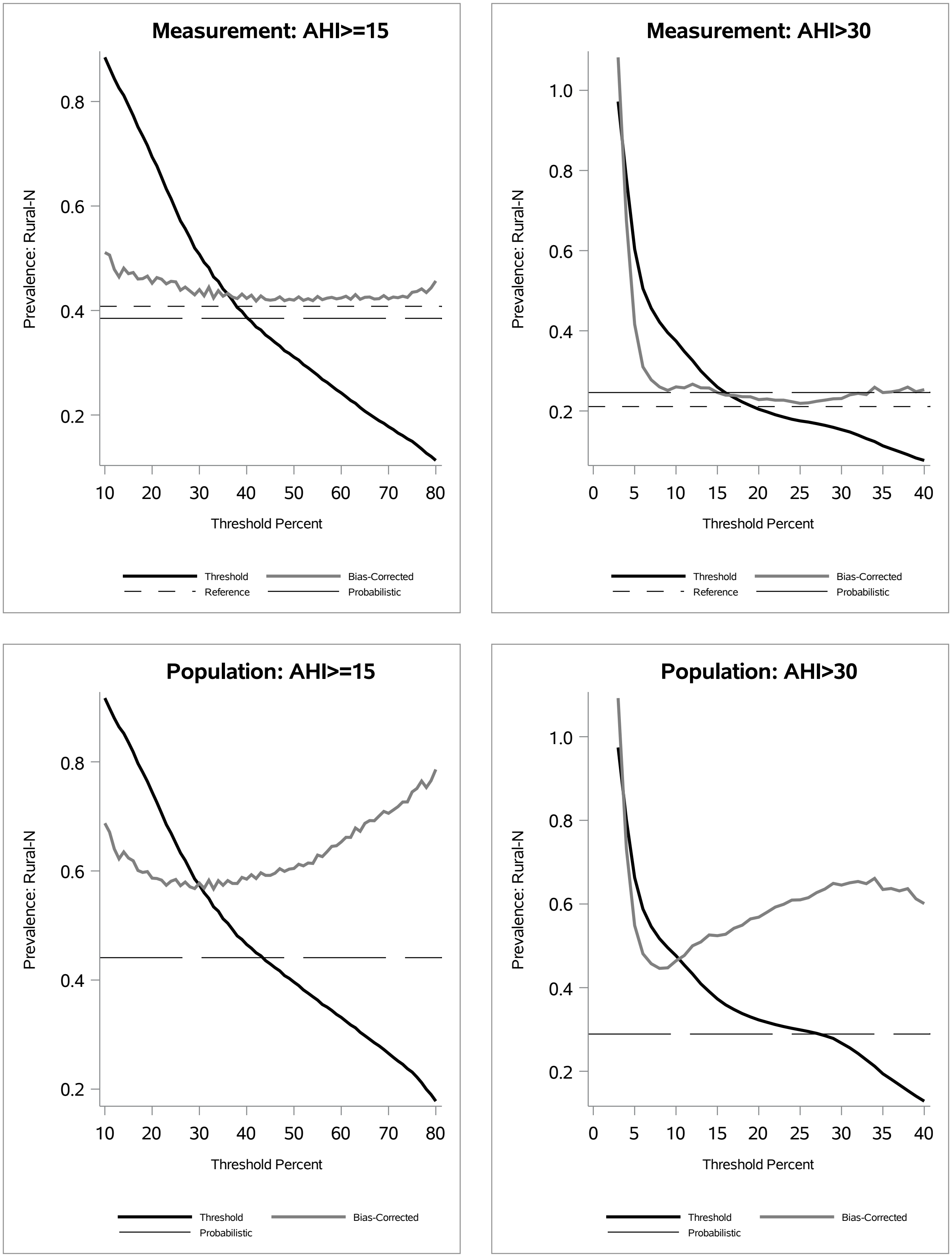
Measurement: AHI>=15
Prevalence: Rural-N
0.2
0.4
0.6
0.8
10 20 30 40 50 60 70 80
Threshold Percent
Threshold
Bias-Corrected
Reference
Probabilistic
Measurement: AHI>30
Prevalence: Rural-N
0.2
0.4
0.6
0.8
1.0
0 5 10 15 20 25 30 35 40
Threshold Percent
Threshold
Bias-Corrected
Reference
Probabilistic
Population: AHI>=15
Prevalence: Rural-N
0.2
0.4
0.6
0.8
10 20 30 40 50 60 70 80
Threshold Percent
Threshold
Bias-Corrected
Probabilistic
Population: AHI>30
Prevalence: Rural-N
0.2
0.4
0.6
0.8
1.0
0 5 10 15 20 25 30 35 40
Threshold Percent
Threshold
Bias-Corrected
Probabilistic

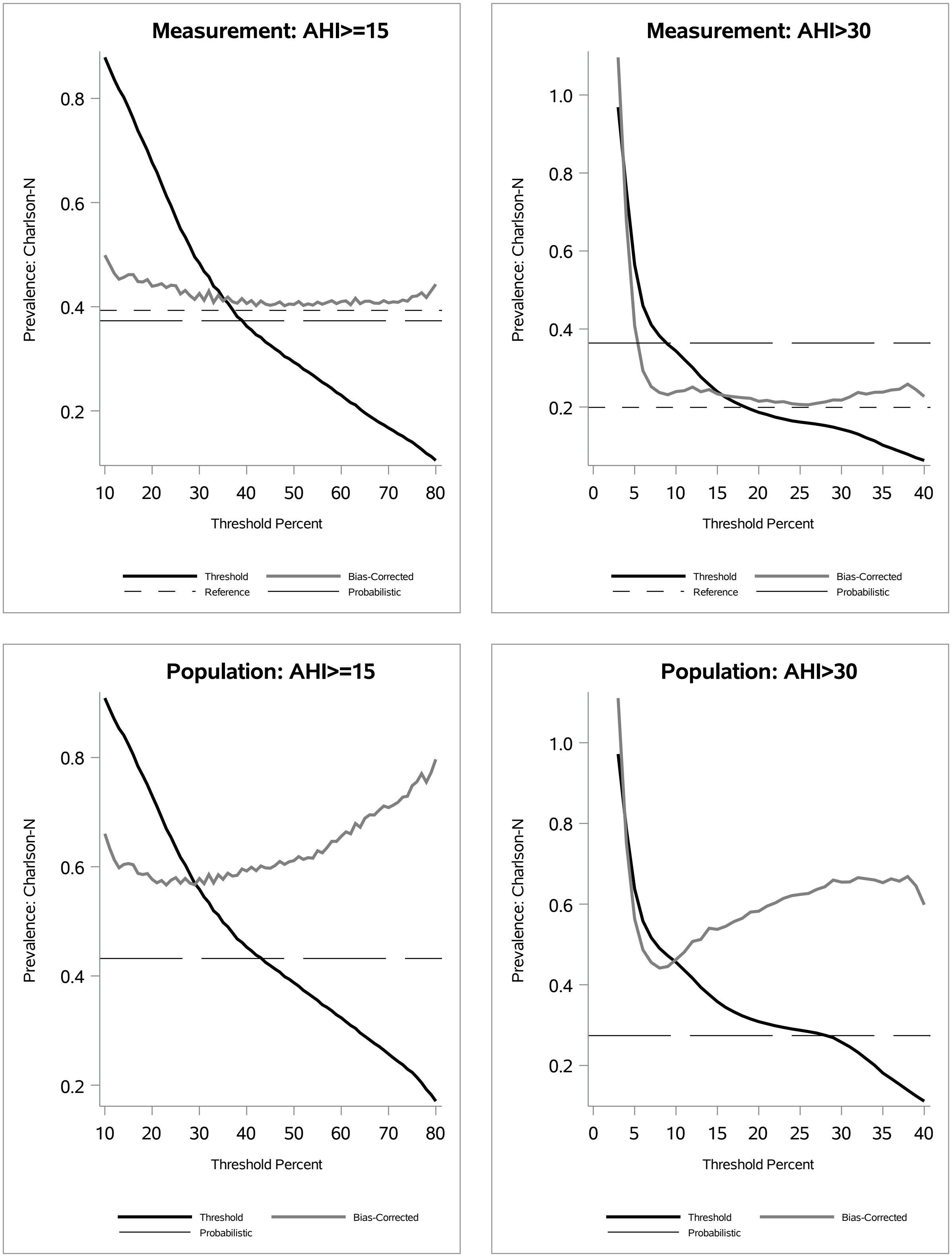

Measurement: AHI>=15
Prevalence: Charlson-N
Threshold Percent
Threshold
Bias-Corrected
Reference
Probabilistic
Measurement: AHI>30
Prevalence: Charlson-N
Threshold Percent
Threshold
Bias-Corrected
Reference
Probabilistic
Population: AHI>=15
Prevalence: Charlson-N
Threshold Percent
Threshold
Bias-Corrected
Probabilistic
Population: AHI>30
Prevalence: Charlson-N
Threshold Percent
Threshold
Bias-Corrected
Probabilistic

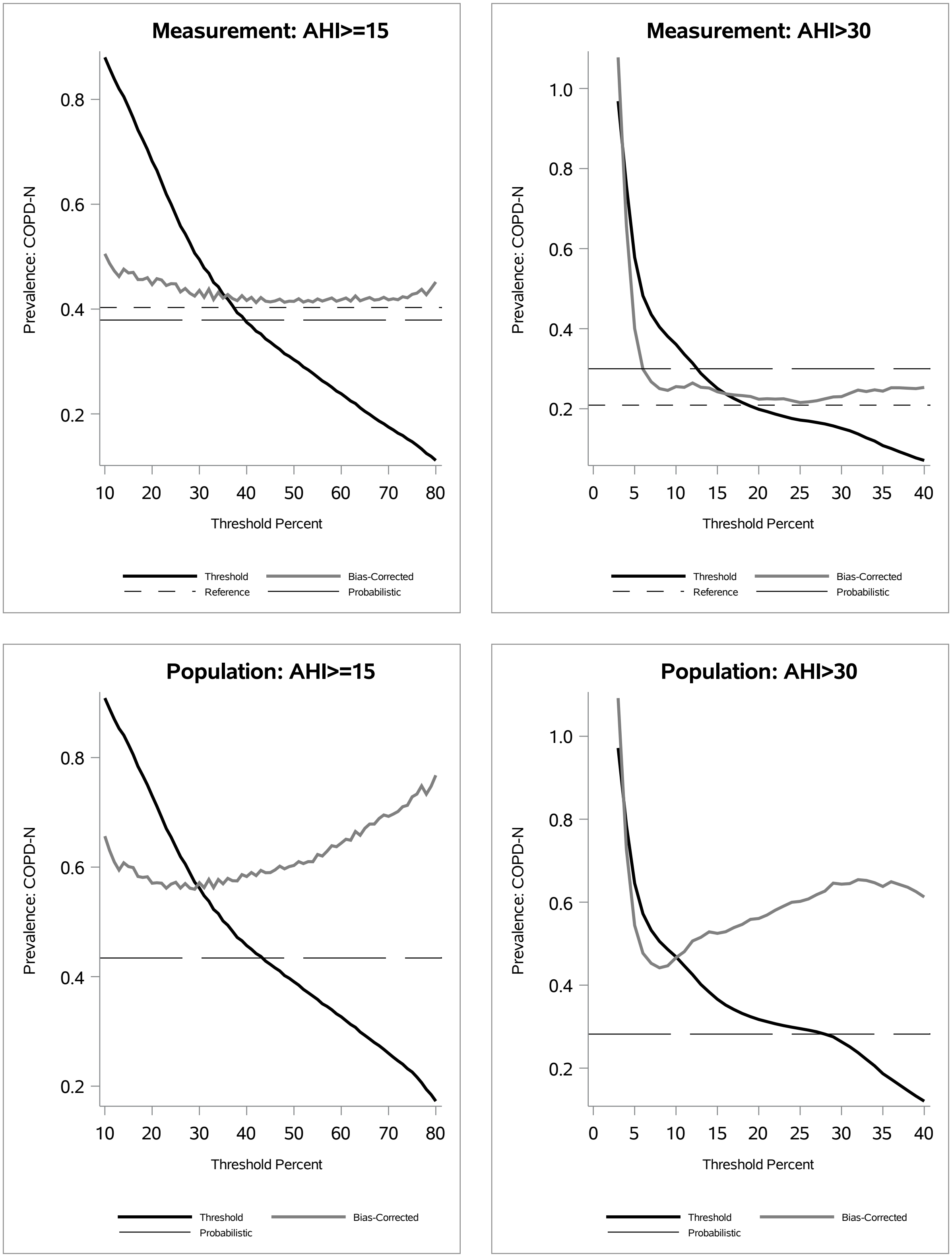

Measurement: AHI>=15
Prevalence: COPD-N
Threshold Percent
Threshold
Bias-Corrected
Reference
Probabilistic
Measurement: AHI>30
Prevalence: COPD-N
Threshold Percent
Threshold
Bias-Corrected
Reference
Probabilistic
Population: AHI>=15
Prevalence: COPD-N
Threshold Percent
Threshold
Bias-Corrected
Probabilistic
Population: AHI>30
Prevalence: COPD-N
Threshold Percent
Threshold
Bias-Corrected
Probabilistic

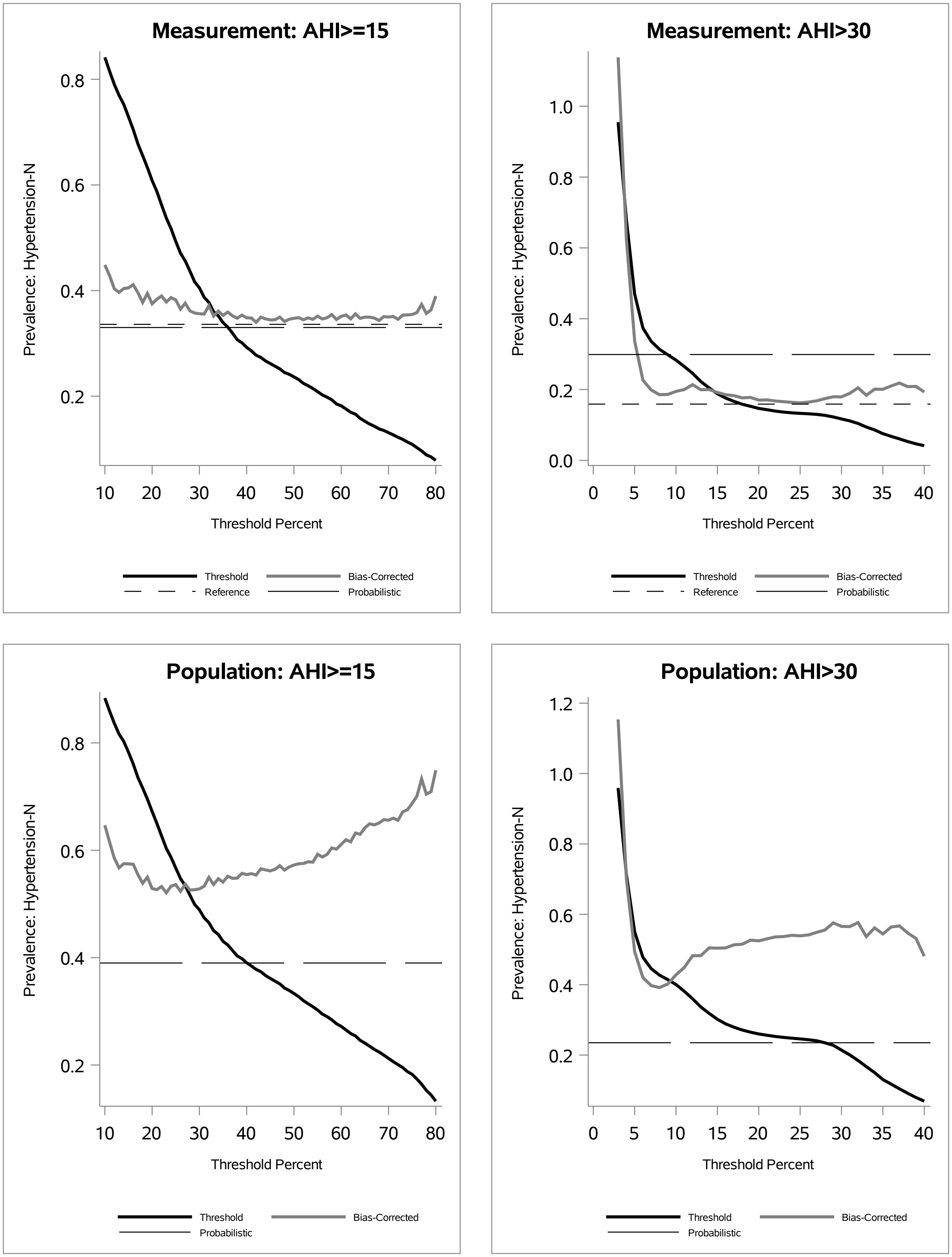
Measurement: AHI>=15
Prevalence: Hypertension-N
Threshold Percent
Threshold
Bias-Corrected
Reference
Probabilistic
Measurement: AHI>30
Prevalence: Hypertension-N
Threshold Percent
Threshold
Bias-Corrected
Reference
Probabilistic
Population: AHI>=15
Prevalence: Hypertension-N
Threshold Percent
Threshold
Bias-Corrected
Probabilistic
Population: AHI>30
Prevalence: Hypertension-N
Threshold Percent
Threshold
Bias-Corrected
Probabilistic

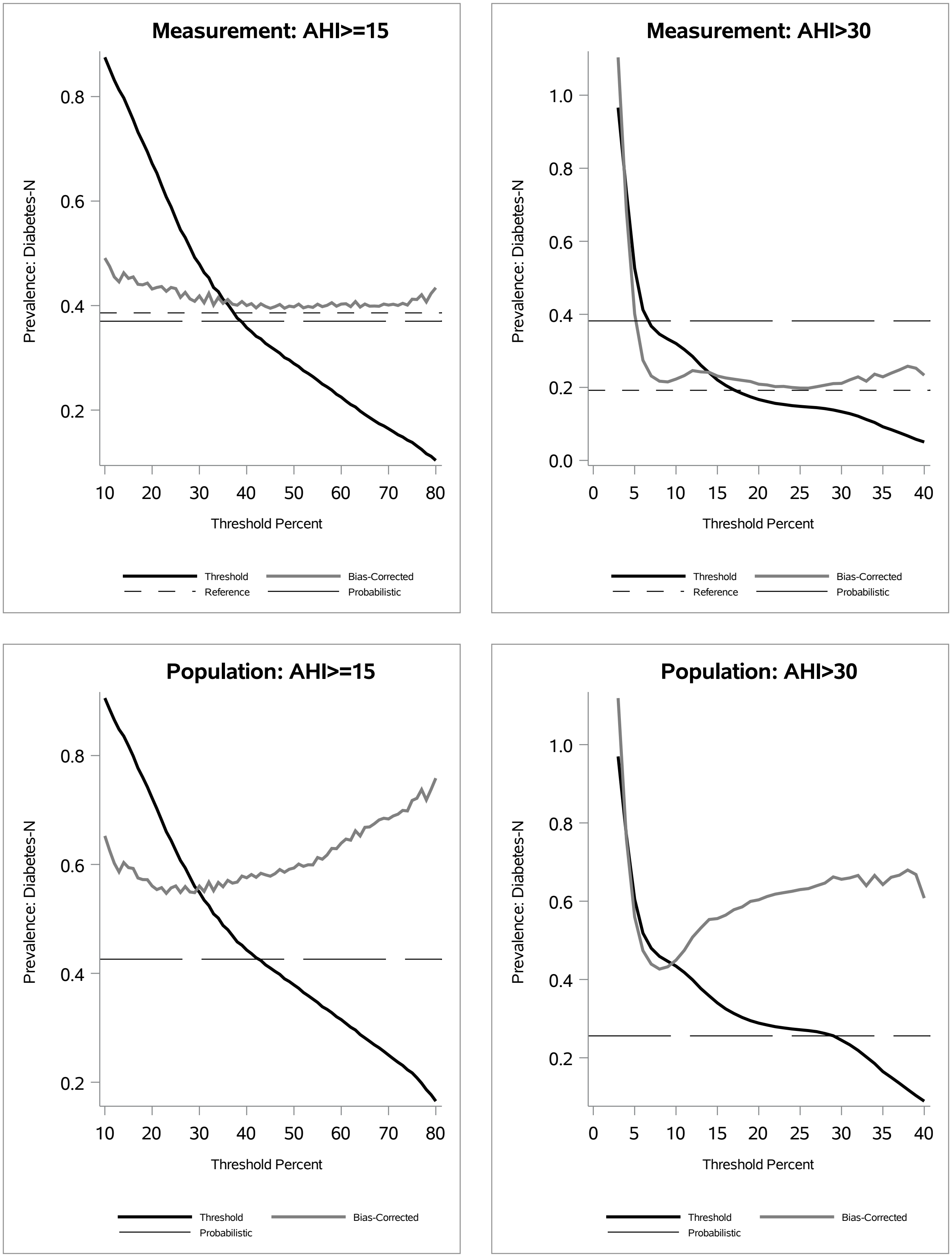
Measurement: AHI>=15
Measurement: AHI>30
Population: AHI>=15
Population: AHI>30
Prevalence: Diabetes-N
Threshold Percent
Threshold
Bias-Corrected
Reference
Probabilistic

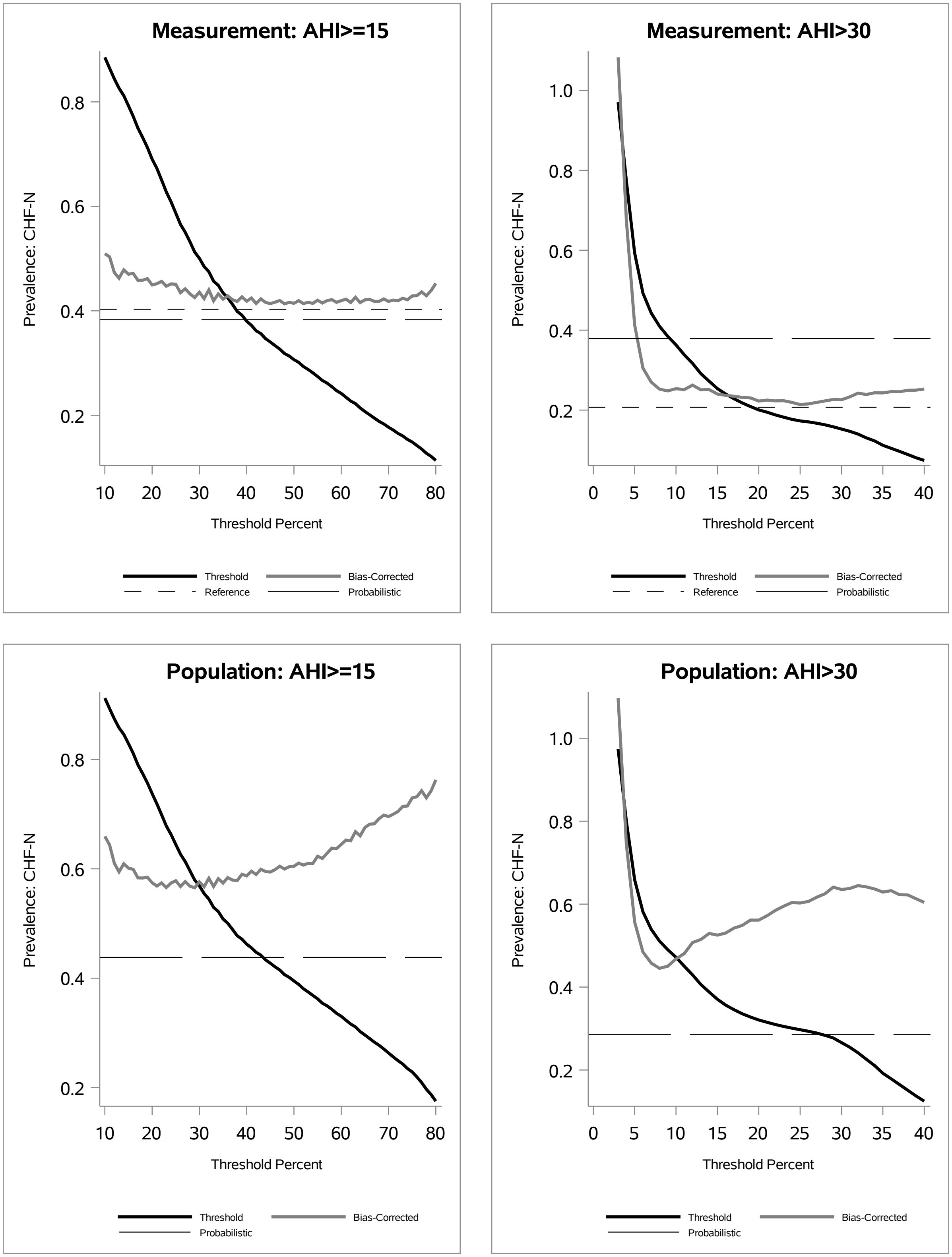
Measurement: AHI>=15
Prevalence: CHF-N
0.2
0.4
0.6
0.8
10
20
30
40
50
60
70
80
Threshold Percent
Threshold
Bias-Corrected
Reference
Probabilistic
Measurement: AHI>30
Prevalence: CHF-N
0.2
0.4
0.6
0.8
1.0
0
5
10
15
20
25
30
35
40
Threshold Percent
Threshold
Bias-Corrected
Reference
Probabilistic
Population: AHI>=15
Prevalence: CHF-N
0.2
0.4
0.6
0.8
10
20
30
40
50
60
70
80
Threshold Percent
Threshold
Bias-Corrected
Probabilistic
Population: AHI>30
Prevalence: CHF-N
0.2
0.4
0.6
0.8
1.0
0
5
10
15
20
25
30
35
40
Threshold Percent
Threshold
Bias-Corrected
Probabilistic

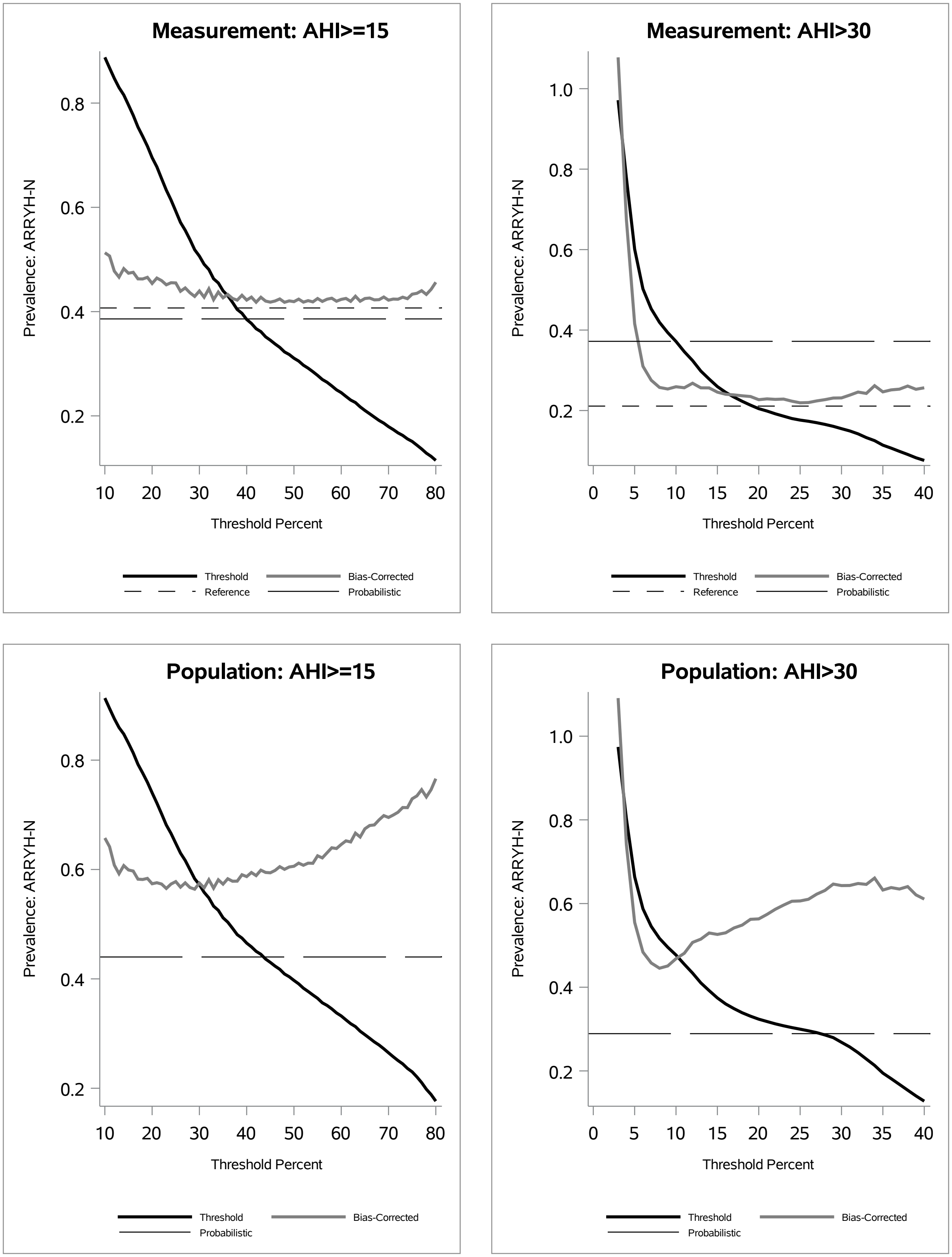
Measurement: AHI>=15
Prevalence: ARRYH-N
Threshold Percent
Threshold
Bias-Corrected
Reference
Probabilistic
Measurement: AHI>30
Prevalence: ARRYH-N
Threshold Percent
Threshold
Bias-Corrected
Reference
Probabilistic
Population: AHI>=15
Prevalence: ARRYH-N
Threshold Percent
Threshold
Bias-Corrected
Probabilistic
Population: AHI>30
Prevalence: ARRYH-N
Threshold Percent
Threshold
Bias-Corrected
Probabilistic

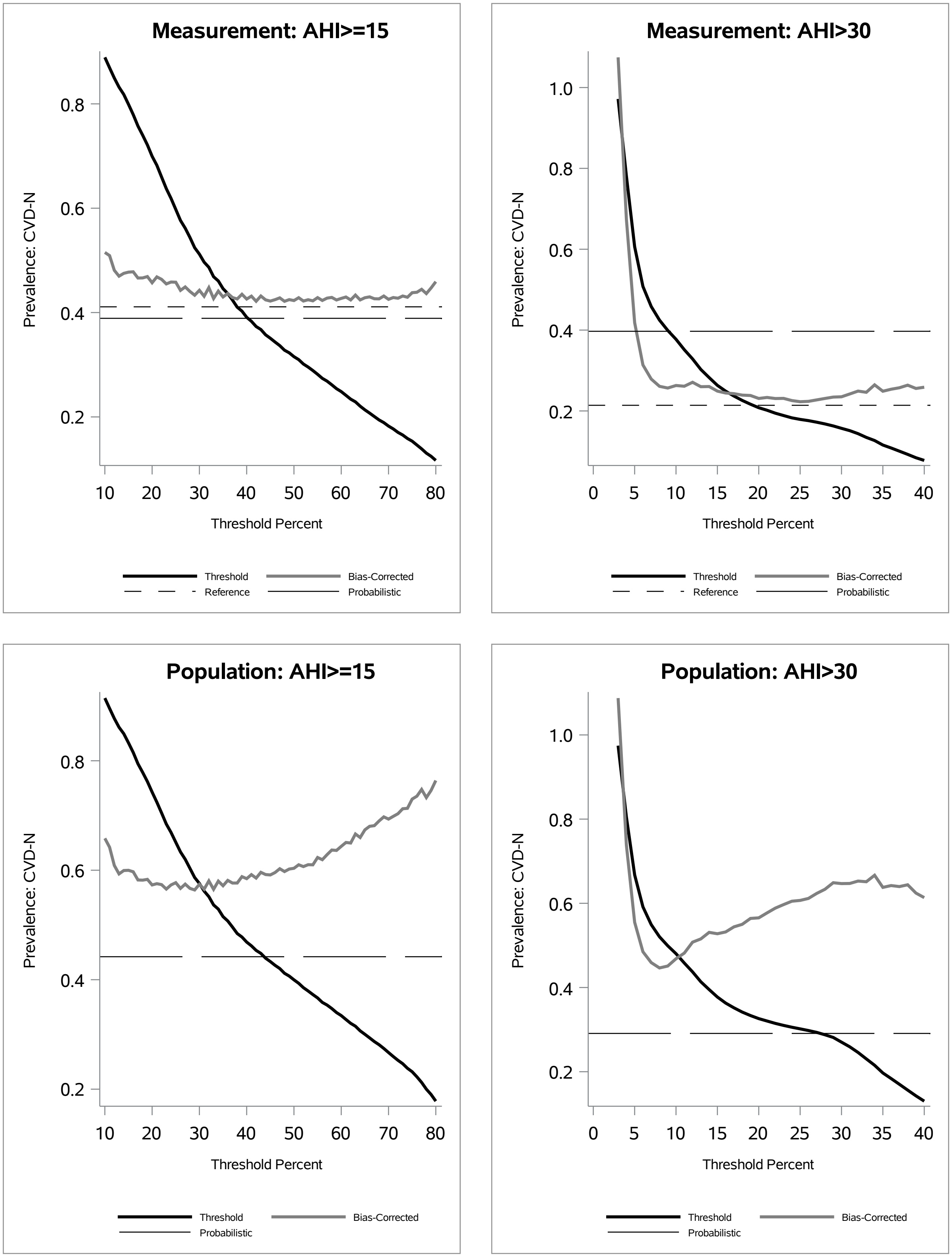
Measurement: AHI>=15
Prevalence: CVD-N
Threshold Percent
Threshold
Bias-Corrected
Reference
Probabilistic
Measurement: AHI>30
Prevalence: CVD-N
Threshold Percent
Threshold
Bias-Corrected
Reference
Probabilistic
Population: AHI>=15
Prevalence: CVD-N
Threshold Percent
Threshold
Bias-Corrected
Probabilistic
Population: AHI>30
Prevalence: CVD-N
Threshold Percent
Threshold
Bias-Corrected
Probabilistic
